# Characteristic chemical probing patterns of loop motifs improve prediction accuracy of RNA secondary structures

**DOI:** 10.1093/nar/gkab250

**Published:** 2021-04-13

**Authors:** Jingyi Cao, Yi Xue

**Affiliations:** School of Life Sciences, Tsinghua-Peking Joint Center for Life Sciences, Beijing Advanced Innovation Center for Structural Biology, Tsinghua University, Beijing 100084, China; School of Life Sciences, Tsinghua-Peking Joint Center for Life Sciences, Beijing Advanced Innovation Center for Structural Biology, Tsinghua University, Beijing 100084, China

## Abstract

RNA structures play a fundamental role in nearly every aspect of cellular physiology and pathology. Gaining insights into the functions of RNA molecules requires accurate predictions of RNA secondary structures. However, the existing thermodynamic folding models remain less accurate than desired, even when chemical probing data, such as selective 2′-hydroxyl acylation analyzed by primer extension (SHAPE) reactivities, are used as restraints. Unlike most SHAPE-directed algorithms that only consider SHAPE restraints for base pairing, we extract two-dimensional structural features encoded in SHAPE data and establish robust relationships between characteristic SHAPE patterns and loop motifs of various types (hairpin, internal, and bulge) and lengths (2–11 nucleotides). Such characteristic SHAPE patterns are closely related to the sugar pucker conformations of loop residues. Based on these patterns, we propose a computational method, SHAPELoop, which refines the predicted results of the existing methods, thereby further improving their prediction accuracy. In addition, SHAPELoop can provide information about local or global structural rearrangements (including pseudoknots) and help researchers to easily test their hypothesized secondary structures.

## INTRODUCTION

Single-stranded RNA molecules fold into intricate secondary structures due to intramolecular base pairing. RNA structures and their rearrangements are intimately involved in a diverse range of cellular processes, including transcription (1,2), alternative splicing (3), translation (4,5) and degradation (6). Although conventional experimental approaches to RNA structure determination, such as crystallography, nuclear magnetic resonance (NMR), and cryogenic electron microscopy (cryo-EM), have been deemed sufficiently accurate, applying these techniques to large or flexible RNAs poses a significant challenge. Recently, multiple enzyme-based (7,8) or chemical-based (9,10) probing experiments have been used to quantitatively determine RNA secondary structures without the limitation of RNA size (10). Selective 2′-hydroxyl acylation analyzed by primer extension (SHAPE) probing (10–12) is one of the most popular methods with the least amount of base-dependent bias (13), and this approach can be implemented in a low-throughput manner by capillary electrophoresis (14) or in a high-throughput manner by next-generation sequencing (15–17). Although next-generation sequencing can probe RNA structures at the whole-transcriptome level, the accuracy is often compromised due to the low read coverage of RNAs (18). This problem is more severe for some long non-coding RNAs that have relatively low expression levels compared with mRNAs.

Generally, RNA residues with higher SHAPE reactivities are more likely to be unpaired, while those with lower SHAPE reactivities tend to be paired. However, the SHAPE reactivities of both paired and unpaired residues can be low (19,20). Moreover, SHAPE data alone cannot directly provide information about two-dimensional (2D) structures, such as pairing partners (21,22). Consequently, SHAPE data are commonly incorporated into RNA secondary structure prediction algorithms as restraints. The minimum free energy (MFE) (23) and the maximum expected accuracy (MEA) (24) are the two main types of algorithms that have been developed; of these, the MFE is the most widely used secondary structure prediction model. SHAPE data are converted into various pseudo-energy terms and serve as restraints in these algorithms, e.g. Fold utility in RNAstructure (12,25), MaxExpect utility in RNAstructure (24), RME (22), RNAsc (26), and RNApbfold (27). These methods require pseudo-energy parameters to combine SHAPE probing data with thermodynamic prediction models, although there is no evidence showing that those combination strategies are mathematically reasonable (28). Given this fact, ‘sample and select’ approaches have been developed. For example, SeqFold (29) selects the centroid structure with the minimal Manhattan distance to SHAPE data, based on the clustering results of Sfold (30). Although SeqFold significantly improves the prediction accuracy when using high-throughput enzymatic probing data, its performance with SHAPE data is not satisfactory compared to pseudo-energy based methods (22,28,29).

Although prediction algorithms with SHAPE restraints effectively increase the accuracy of the predicted structures, there remains space for improvement (31). Overall, there is a false negative and false discovery rate for shorter RNAs (<∼150 nt) of approximately 20% (32,33), and the prediction accuracy decreases dramatically for longer RNAs (34). More importantly, the performance of these algorithms in identifying high-level interactions (e.g. pseudoknot) and dynamic structural changes is far from satisfactory. As the prediction accuracy of methods using different pseudo-energy parameters has reached a ‘ceiling’ (35), greater effort should be made to exploit the various types of structural information encoded in SHAPE signals. For example, patteRNA uses a Gaussian mixture model-hidden Markov model (GMM-HMM) to search for user-defined RNA structural motifs from SHAPE profiling data (36). However, patteRNA aims neither to identify characteristic SHAPE patterns for structural motifs nor to predict the most optimal structures for given SHAPE data. Instead, it is designed to automatically mine all putative locations for target motifs; thus, the false discovery rate of patteRNA-recognized loops is comparatively high (36).

To bridge the gap between SHAPE data and structural information, we developed a computational method, SHAPELoop, which establishes direct relationships between SHAPE patterns and loop motifs of various types and lengths. Equipped with such relationships, SHAPELoop quantitatively estimates the discrepancies between the SHAPE reactivities of predicted loops and the characteristic SHAPE patterns, and selects the candidate structure most consistent with the characteristic SHAPE patterns. The application of SHAPELoop to benchmark RNAs demonstrates that SHAPELoop outperforms the commonly used pseudo-energy-based and ‘sample and select’-based prediction models. Moreover, the design principles of SHAPELoop render it an effective tool to adequately decipher SHAPE-implied structural information, which can potentially inform us of local or overall structural rearrangements (including pseudoknots) mediated by varied folding conditions or mutated primary sequences.

## MATERIALS AND METHODS

### Benchmark RNAs and their SHAPE probing data

To identify the characteristic SHAPE patterns for hairpin, internal, and bulge loop motifs, we analyzed a dataset consisting of 11 RNAs with known structures as determined by crystallography or NMR and with low-throughput SHAPE reactivities publicly available (12,37–39) (Supplementary Table S1). These RNAs include nine short RNAs and two long rRNAs (*Escherichia coli* 16S and 23S). The two long rRNAs were divided into 10 domains for structural prediction following the published strategies (40,41).

### Identification of characteristic SHAPE patterns

**Figure 1.**
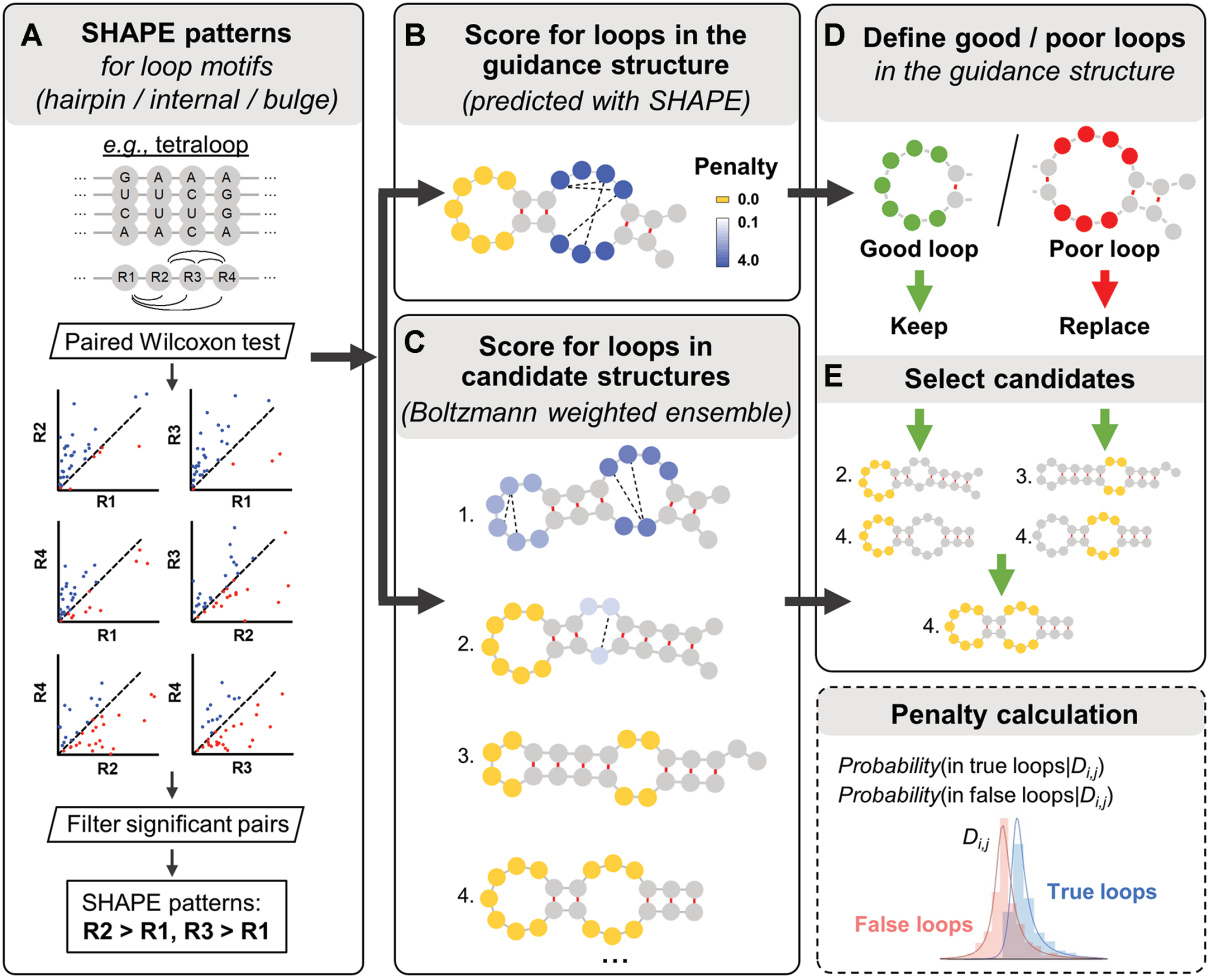
SHAPELoop workflow. (**A**) SHAPELoop is established based on the characteristic patterns of selective 2′-hydroxyl acylation analyzed by primer extension (SHAPE) for loop motifs of various types (hairpin, internal, and bulge) and lengths. The difference in SHAPE reactivities between any two positions of each loop motif is tested, and pairwise comparison results with sufficient significance are defined as the characteristic SHAPE patterns. The next two major steps of SHAPELoop are calculating penalties for loops in the guidance (minimum free energy, MFE) structure (**B**) and the Boltzmann-weighted candidate ensemble (**C**). Discrepancies between the SHAPE reactivities of predicted loops and the characteristic SHAPE patterns are estimated using a posterior probabilistic model and then used to calculate the penalties for predicted loops. (**D**) Loops in the guidance structure are divided into ‘good’ and ‘poor’ loops based on their penalties. (**E**) Candidates that retain all ‘good’ loops and have loops with lower penalties in ‘poor’ loop regions will be selected as the predicted structures.

Hairpin, internal, and bulge loops with two flanking base pairs were extracted from the benchmark RNAs (Table 1). Loops involved in pseudoknot structures were removed. A loop motif was defined as any loops with the same length and type (hairpin, bulge, or internal), irrespective of their sequences. We applied paired Wilcoxon signed-rank tests (42) to detect significant differences in SHAPE reactivities between any two positions in each loop motif (Figure 1A). Among the pairs under comparison, if the *P*-value was <0.05 and was ranked as the top two smallest *P*-values for that loop motif, the pair was selected as a characteristic SHAPE pattern of that specific motif (Supplementary Figure S1). We also attempted to identify SHAPE patterns for multi-branch loops; however, such data were too diverse for a statistical analysis (Supplementary Table S2).

**Table 1.** Summary of the loops extracted from benchmark RNAs

	Total number	Number of motif type	Length range
Hairpin	106	13	2–13, 15
Bulge	33	6	2–7
Internal	87	25	2–11, 13, 14, 16, 23
Multi-branch	60	57	1–15, 18, 19, 22, 25, 27

### Statistics of sugar conformation

RNA three-dimensional (3D) structures were obtained from the Protein Data Bank (PDB). The non-redundant list of PDB structures was downloaded from the Nucleic Acid Database (v3.127) with the resolution threshold set to 3.0 or 4.0 Å, resulting in 966 and 1475 structures, respectively. Secondary structure information of the selected PDB structures was derived from the outputs of DSSR software (v1.6.9) (43). The sugar conformations of loop residues were determined as C2′-endo or C3′-endo based on whether both of the following two criteria were satisfied: (i) the sugar pucker is C2′-endo as determined by DSSR and (ii) 2′-OH forms a hydrogen bond with pyrimidine O2, purine N3, or the non-bridging O from the phosphodiester. These three types of hydrogen bonds were reported to be associated with the C2′-endo-conformation and high SHAPE reactivities (44–46).

### SHAPELoop framework

SHAPELoop is designed to refine loops in the MFE structures (referred to as ‘guidance structures’ hereinafter) to make them agree better with the characteristic SHAPE patterns. To this end, SHAPELoop includes five major steps: (i) identifying characteristic SHAPE patterns (Figure 1A); (ii) scoring loops in the guidance structure (Figure 1B); (iii) scoring loops in the candidate structures (Figure 1C); (iv) classifying loops in the guidance structure (Figure 1D); and (v) selecting a final structure from the candidates (Figure 1E). For an RNA of interest, SHAPELoop first identifies the characteristic SHAPE patterns with a ‘leave-one-out’ strategy; that is, the RNA being predicted is removed from the benchmark dataset to avoid biased evaluation. Second, SHAPELoop generates the guidance structure and candidate ensemble. The guidance structure is the MFE structure predicted by the RNAstructure-Fold algorithm with SHAPE restraints (12). Meanwhile, 1000 candidates are sampled using the partition and stochastic utilities in RNAstructure, with both the Boltzmann conditional probabilities (30) and SHAPE restraints considered. To score loops in these structures rigorously, SHAPELoop employs a probabilistic model to quantitatively estimate the degree of difference between the SHAPE patterns of the predicted loop and the characteristic SHAPE patterns, as detailed below.

First, the true or false loops are defined as loops that are either present or absent in the reference structures (the pairs of bases mentioned below refer only to the bases involved in characteristic SHAPE patterns). When the experimental SHAPE difference between a pair of bases is given, a posterior probabilistic model is used to calculate their probability of belonging to a true loop, i.e. *P*(*W_i,j_* = 1*|D_i,j_*), where *D_i,j_* is the SHAPE difference between the pair of bases *i* and *j*, and *W_i,j_* is defined as the structure class, *W_i,j_*${\rm{\;}} \in {\rm{\;}}$(0,1) (1 = the pair of bases resides in a true loop, 0 = the pair of bases resides in a false loop). Similar to the models used in other studies (22,36), *P*(*W_i,j_ =* 1*|D_i,j_*) can be calculated as below:

$$\begin{eqnarray*}&&P(\;{W_{i,j}} = \;1|{D_{i,j}}) \nonumber\\ &&\quad= \frac{{P({D_{i,j}}|{W_{i,j}} = 1) \cdot P\left( {{W_{i,j}} = 1} \right)}}{{P({D_{i,j}}|{W_{i,j}} = 1) \cdot P\left( {{W_{i,j}} = 1} \right) + P({D_{i,j}}|{W_{i,j}} = 0) \cdot P\left( {{W_{i,j}} = 0} \right)}} \end{eqnarray*}$$

In this equation, *P*(*W*) is the prior probability that a pair of bases belongs to a specific structure class, which can be estimated as the fraction of pairs of bases residing in true or false loops extracted from the candidate ensemble of benchmark RNAs. In our study, *P*(*W_i,j_ =* 1) = 0.549, *P*(*W_i,j_ =* 0) = 0.451. *P*(*D|W*) is the probability of observing a certain SHAPE difference *D* between the pair of bases, given a structure class; this can be estimated from the SHAPE data of reference structures through maximum likelihood fitting. We used a normal-inverse Gaussian distribution to fit the distribution of the SHAPE difference between pairs of bases belonging to true loops (*W_i__,j_ =* 1) and a Johnson's SU distribution for false loops (*W_i,j_ =* 0). Both distributions passed the Kolmogorov–Smirnov test for goodness-of-fit. We used the *norminvgauss* and *johnsonsu* functions from the python module *scipy.stats* to fit these two distributions (Supplementary Figure S2). Inspired by the logarithm of posterior odds used in other models (22,47), we defined a penalty score for each predicted loop, denoted as $\mathop \sum \limits_1^N {\delta _{i,j}}$, where *N* is the total number of pairs of bases that satisfy *P*(*W_i__,j_=* 1*|D_i,j_*) < 0.5, and $\;{\delta _{i,j}}{\rm{\;}}$is calculated as below:

$$\begin{equation*}\;{\delta _{i,j}} = \; - \ln \frac{{P(\;{W_{i,j}} = \;1|{D_{i,j}})}}{{1 - P(\;{W_{i,j}} = \;1|{D_{i,j}})}}\end{equation*}$$

After obtaining the penalty scores, loops in the guidance structure are divided into ‘good’, ‘fair’, and ‘poor’ groups based on their penalties (‘good’: penalty = 0; ‘fair’: 0 $ <$ penalty $ \le$ 1 or characteristic SHAPE patterns are unavailable; ‘poor’: penalty > 1). In the last step, SHAPELoop selects the candidate that satisfies the following criteria: (i) all ‘good’ loops are retained; (ii) there are no loops with penalties higher than 1 in ‘fair’ loop regions; (iii) ‘poor’ loops are replaced by loops with lower penalties; and (iv) the total penalty is the lowest among the candidates that meet the three criteria mentioned above. If no candidate satisfies all these four criteria, the one with the lowest penalty will be selected. Please note that here we define a loop region as the loop itself, along with the four preceding nucleotides and the four following ones. The centroid structure can be selected as the predicted secondary structure if suboptimal structures are not desired (29,30).

### MC-Fold candidate ensemble

MC-Fold was used to generate a candidate ensemble by considering both non-canonical base pairs and SHAPE restraints (48). SHAPE reactivities were converted into probabilities of being unpaired using a linear model included in RNAfold (a slope of 0.68 and an intercept of 0.2) (49), and then divided into ‘high reactivity’ (probability > 0.85) and ‘medium reactivity’ (0.65 $ <$ probability $ \le$ 0.85) groups according to the input requirements of MC-Fold.

### Robustness test

The effect of choosing a different guidance structure on the prediction results was examined by substituting the MFE structure with other suboptimal secondary structures predicted by the RNAstructure-Fold algorithm with SHAPE restraints. We applied three statistical metrics to evaluate the performance of these guidance structures: sensitivity, positive predictive value (PPV), and Matthews correlation coefficient (MCC). Sensitivity is defined as the fraction of the base pairs in the native structures that are correctly predicted, and PPV is defined as the fraction of the predicted base pairs that occur in the native structures. The RNAstructure-scorer algorithm was used to calculate these two metrics. MCC, which summarizes both sensitivity and PPV, was calculated following a published method (33). The robustness of SHAPELoop to noise in SHAPE data was also inspected. To this end, we randomly selected increasing fractions of data points and shuffled their SHAPE reactivities to simulate noise in the SHAPE data. The guidance structure and candidate ensemble were re-generated using the shuffled SHAPE reactivities. This procedure was repeated ten times for each noise level, and the average sensitivity and PPV were calculated on benchmark RNAs each time.

## RESULTS

### SHAPELoop identifies characteristic SHAPE patterns for loop motifs

**Figure 2.**
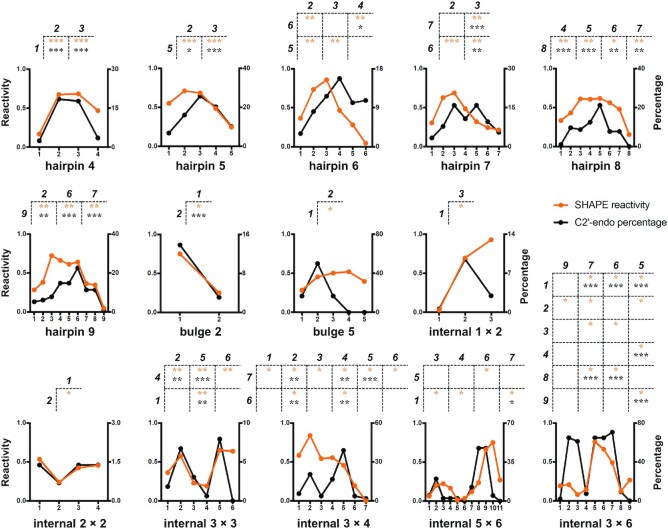
Characteristic SHAPE patterns for hairpin, internal, and bulge loop motifs. The averaged SHAPE reactivities at all positions of each loop motif in benchmark RNAs are shown by orange lines. The proportions of the C2′-endo sugar pucker conformation (for RNAs in the non-redundant PDB list with 4.0 Å cutoff) are shown by black lines. The characteristic SHAPE patterns of each loop motif are shown at the top of each subplot, with the residues at the top of each table showing higher SHAPE reactivities than the residues to the left, and their *P*-values are indicated by asterisks (*0.01 < *P*-value ⩽ 0.05; **0.001 < *P*-value ⩽ 0.01; ***P-value ⩽ 0.001). The residue position is counted from the 5′ end of each loop motif. Detailed *P*-values and sample sizes are listed in Supplementary Tables S3 and S4.

We identified the characteristic SHAPE patterns for loop motifs to establish relationships between SHAPE reactivities and secondary structure features. To this end, we first extracted hairpin, internal, and bulge loops from benchmark RNAs (12,37–39) (Supplementary Table S1) and classified them into different loop motifs based on their types and lengths (Table 1). Then, for each loop motif, the difference in SHAPE reactivities between any two positions (*D_i,j_*) was tested, and pairwise comparison results with sufficient significance were defined as SHAPE patterns that were characteristic of the specific loop motif (see Materials and Methods). As a result, we obtained characteristic SHAPE patterns for six hairpin loop motifs (4–9 nt), two bulge loop motifs (2 nt and 5 nt), and six internal loop motifs (1 × 2 nt, 2 × 2 nt, 3 × 3 nt, 3 × 4 nt, 3 × 6 nt, and 5 × 6 nt) (Figure 2 and Supplementary Tables S3 and S4). The *D_i,j_* distributions of these loop motifs are markedly more concentrated above zero than those of false loops (i.e. loops extracted from the candidate ensemble and not present in reference structures, as defined in Materials and Methods) (Supplementary Figure S3), suggesting that these SHAPE patterns can be used to evaluate loop motifs in predicted secondary structures.

To bolster the rationality of these characteristic SHAPE patterns, we examined whether these patterns reflect specific 3D structural features of each loop motif. As SHAPE reagents acylate the 2′-OH group of RNA ribose, we speculated that ribose puckering might be an underlying structural factor that dictates SHAPE patterns. The sugar conformations of nucleotides in an RNA A-form helix are dominated by C3′-endo. In contrast, nucleotides with a C2′-endo sugar pucker are often associated with greater conformational flexibility and local motions (50–53). The difference in C3′-endo/C2′-endo sugar conformation between the two positions involved in each characteristic SHAPE pattern was subjected to the paired Wilcoxon signed-rank test. The results show that, for most of the characteristic SHAPE patterns, the trends of SHAPE reactivities are consistent with the preferences of the C2′-endo sugar pucker (Figure 2, Supplementary Figure S4, and Supplementary Tables S3 and S4). In other words, residues with C2′-endo sugar conformations are more likely to have high SHAPE reactivities. This finding agrees with previous studies showing that nucleotides with a C2′-endo sugar pucker have lower activation barriers for the 2′-OH acylation reaction (46) and are more reactive to SHAPE reagents (44,45,54). These results indicate that loops with the same loop motif share similar structural features, particularly the sugar pucker preference at specific positions. Therefore, it is both possible and reasonable to extract characteristic SHAPE patterns for loop motifs.

We further confirmed the reliability of the sugar conformation patterns by reviewing the available literature on tetraloops, the most characterized loop motif. Among all reported tetraloops, GNRA (55) and UNCG (56) (N = any and R = G/A) are the most abundant species. The second and third residues in a UNCG loop are C2′-endo, whereas the first and fourth residues are C3′-endo (57–59). Similarly, the second and third residues in GNRA loops favor a mixture of C3′-endo and C2′-endo, whereas the first and fourth residues exist only in C3′-endo (57,60–63). In addition to GNRA and UNCG, other tetraloops, such as AGAA, UUUC, GGAG and CAAC, are also more likely to adopt a C2′-endo conformation at the second and third residues than the first residue (58,64–67). These published results support the sugar conformation patterns that we identified for the tetraloop motif and are also accordant with their characteristic SHAPE patterns (the second and third positions are higher than the first position, as shown in Figure 2). Additionally, we found that loops extracted from benchmark RNAs were also highly abundant in RNA structures deposited in PDB (Supplementary Figures S5 and S6). Collectively, these results demonstrate that the characteristic SHAPE patterns identified on benchmark RNAs are both representative and structurally explainable.

### SHAPELoop effectively identifies loops that are falsely predicted

**Figure 3.**
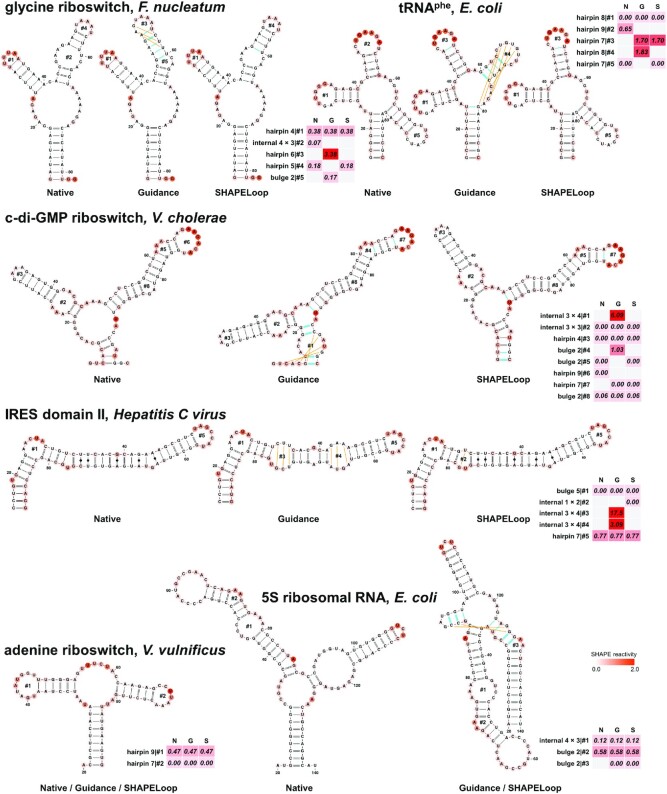
SHAPELoop efficiently identifies loops that are falsely predicted by RNAstructure and selects candidates with low penalties. Shown are the native structures (N), RNAstructure-predicted guidance structures (G), and SHAPELoop-predicted structures (S) for six benchmark RNAs. The SHAPE reactivities are shown on the structures using a coloring scheme. The penalties are shown as heat maps. The false-positive (incorrect) base pairs in the guidance or SHAPELoop-predicted structures are shown by green lines, and false-negative (missing) base pairs are shown by orange lines. The remaining benchmark RNAs are shown in Supplementary Figures S7 and S8.

We developed an RNA secondary structure prediction tool, SHAPELoop (see Materials and Methods), using the identified characteristic SHAPE patterns for loop motifs. Before evaluating SHAPELoop-predicted structures, we first examined whether SHAPELoop could identify incorrectly predicted loops in the guidance structures. Among the nine short RNAs and 10 domains of the long *E. coli* 16S and 23S rRNAs, SHAPELoop identified ‘poor’ loops (penalty > 1) in the guidance structures of five short RNAs and eight domains, and these ‘poor’ loops were indeed falsely predicted (Figure 3 and Supplementary Figures S7 and S8). In contrast, the penalties for loops in native structures were much lower. For the remaining RNAs and domains, falsely predicted loops that were not categorized as ‘poor’ loops generally lacked characteristic SHAPE patterns. For example, the worst predicted loop region in the guidance structure of *E. coli* 5S rRNA included a 1 × 1 nt internal loop and a 3 nt hairpin loop, but no characteristic SHAPE patterns were identified for these two loop motifs (Figure 3). Similar phenomena were observed on *Saccharomyces cerevisiae* P546 domain (a 1 × 9 nt internal loop), *E. coli* 16S rRNA domain 4 (a 3 nt hairpin loop and a three-way junction), and *E. coli* 23S rRNA domain 6 (1 × 1 nt, 2 × 3 nt, and 2 × 6 nt internal loops and a four-way junction). In summary, these results suggest that characteristic SHAPE patterns identified by SHAPELoop can reliably detect falsely predicted loops.

### SHAPELoop significantly improves the accuracy of RNA secondary structure prediction

Given the finding that characteristic SHAPE patterns are useful for distinguishing falsely predicted loops from correctly predicted loops, we proceeded to examine whether SHAPELoop could improve the accuracy of RNA secondary structure prediction. For the five short RNAs whose ‘poor’ loops were successfully identified by SHAPELoop, the ‘poor’ loops were replaced by loops with lower penalties, and the resulting structures were indeed more accurate than the guidance structures (Figure 3, Supplementary Figure S7, and Supplementary Table S5). Of the 10 domains of *E. coli* 16S and 23S rRNAs, the accuracy of six domains was improved by SHAPELoop, although with only modest improvements in some domains (Supplementary Figure S8 and Supplementary Table S5). The inadequate sampling of candidates is one important reason for these limited improvements. For example, falsely predicted loops in the guidance structures of *E. coli* 23S rRNA domains 3 and 5 were successfully identified by SHAPELoop; however, these ‘poor’ loops were not replaced with ‘good’ ones in the final selected structures due to the absence of the correct structure in the candidate ensemble (Supplementary Figure S8). Unsurprisingly, SHAPELoop selected the correct structures after the reference structures were manually added to the candidate ensemble. Another important reason for the insufficient improvement is the lack of characteristic SHAPE patterns for complex structural motifs. Indeed, we observed worse predictions of loop motifs lacking characteristic SHAPE patterns, particularly for multi-branch loops (Supplementary Figure S9). Undoubtedly, the increasing availability of SHAPE data for these complex structural motifs will help to identify characteristic SHAPE patterns for them and eventually improve the prediction accuracy of long RNAs.

Notably, the characteristic SHAPE patterns are not limited to evaluations of structures containing only canonical base pairs and G-U wobbles. In fact, if the penalty for one loop is considerably high but no better candidates can be selected, there is a possibility that the RNA forms some non-canonical base pairs. Considering that most RNA secondary structure prediction tools, including RNAstructure, cannot predict non-canonical base pairs, we did not take these into account initially. However, for the *Hepatitis C virus* IRES domain II, the loop penalties for its guidance structures were much higher than for those of other RNAs, and SHAPELoop failed to select structures with lower penalties from the candidate ensemble; therefore, we speculated that this RNA might include non-canonical base pairs. In line with this, we reconstructed the candidate ensemble using MC-Fold (48) to include non-canonical base pairs, and as a result, we found that SHAPELoop preferred non-canonical base pairs to loops in the ‘poor’ loop regions (Figure 3). Indeed, the native structure forms five non-canonical base pairs in the same regions. These non-canonical base pairs mimic the A-form dsRNA structure and are essential for the activation of the protein kinase PKR (68,69). These results demonstrate that the application of SHAPELoop can be extended to predictions of structures with non-canonical base pairs.

**Figure 4.**
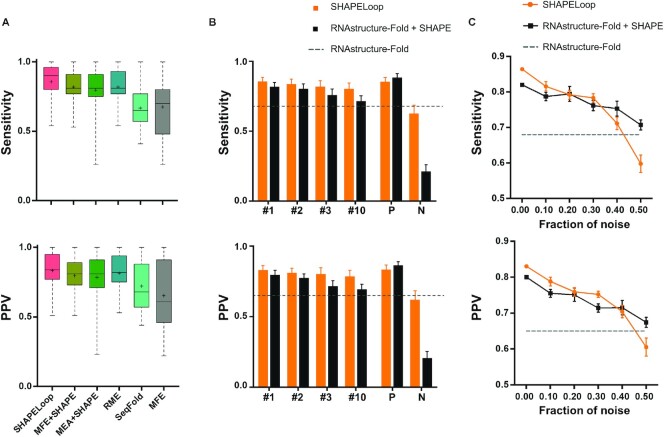
Performance comparison between SHAPELoop and other algorithms. (**A**) Boxplot comparison of the performances of SHAPELoop and five other predictors on benchmark RNAs. The central marks indicate the medians, and the bottom and top edges of the boxes indicate the 25th and 75th percentiles, respectively. The mean values are shown as ‘+’. (**B**) Comparison of the performances of SHAPELoop using different guidance structures on benchmark RNAs. The guidance structures are replaced by structures of different energy rankings: ‘#1’, ‘#2’, ‘#3’, and ‘#10’ refer to the structure with the first (MFE), second, third, and tenth lowest energy, respectively. ‘P’ (i.e. positive) indicates the RNAstructure-predicted candidates with the highest Matthews correlation coefficient (MCC), and ‘N’ (i.e. negative) indicates the RNAstructure-predicted candidates with the lowest MCC. (**C**) The sensitivity and positive predictive value (PPV) yielded by SHAPELoop and RNAstructure-Fold, with increasing fractions of SHAPE data replaced by randomized values. Error bars in (B) and (C) represent the standard errors of the means.

Overall, SHAPELoop achieved the highest mean and median sensitivity and PPV, compared with RNAstructure-Fold (12), RNAstructure-MaxExpect (24), RME (22), and SeqFold (29) (Figure 4A and Supplementary Table S5). The difference between SHAPELoop and the second-best predictor RME was statistically significant in terms of sensitivity but not PPV (paired Wilcoxon signed-rank test *P*-value = 0.024 and 0.188, respectively), and the improvement with SHAPELoop relative to the guidance structures (predicted by RNAstructure-Fold) was statistically significant in terms of both sensitivity and PPV (*P*-value = 0.010 and 0.005, respectively).

### SHAPELoop is moderately robust to variations in guidance structures and noise in SHAPE probing data

Given that SHAPELoop requires a guidance structure of the RNA being predicted, we examined the robustness of SHAPELoop against alternative guidance structures. In doing so, we replaced the MFE structure with other structures, such as those with the 2nd, 3rd or 10th lowest free energy. The mean sensitivity and PPV of the MFE structure (without SHAPE restraints) on benchmark RNAs served as the baseline values. As a result, the SHAPELoop-predicted structures outperformed the guidance structures in all cases (Figure 4B). To further explore the upper and lower limits of SHAPELoop prediction, we calculated the MCC for each structure predicted by RNAstructure-Fold (with SHAPE restraints) and selected the highest or lowest MCC structure as the guidance structure for each benchmark RNA. The results show that SHAPELoop-predicted structures remained superior to the lowest-MCC guidance structures; however, there was no improvement in comparison with the highest-MCC guidance structures (Figure 4B), which could be attributed to the inadequate representation of sampled candidates and the lack of characteristic SHAPE patterns for intricate loop motifs. In addition, noise in the SHAPE probing data is another potential cause of the insufficient improvement observed.

Next, we assessed the performance of SHAPELoop under different levels of noise in SHAPE probing data. To introduce noise into SHAPE data, we randomly selected increasing fractions of data points and shuffled the SHAPE reactivities at these data points (29). The guidance structure and candidate ensemble were both re-generated using the shuffled SHAPE reactivities. The results show that the mean sensitivity and PPV of SHAPELoop outperformed RNAstructure-Fold when the fraction of noise was <20%, and the performance of SHAPELoop prediction was evidently inferior to that of RNAstructure-Fold when the noise fraction increased to 40% (Figure 4C). In summary, the results indicate that SHAPELoop is moderately resistant to noise in SHAPE probing data. Despite this, special care needs to be taken to follow the standard SHAPE procedure, thereby improving the quality of SHAPE data and making the SHAPE pattern identification and structure modeling procedures more reliable.

### SHAPELoop penalty helps to identify pseudoknots in RNA structures

The kissing loop, also called the loop-loop pseudoknot (70,71), is one type of RNA tertiary interaction. The formation of a kissing loop involves the transition of residue states from single-stranded to paired. Given that the characteristic SHAPE patterns were identified for loops that are not engaged in pseudoknot interactions, in principle, penalties calculated based on these SHAPE patterns can help us to test hypotheses regarding kissing loops.

**Figure 5.**
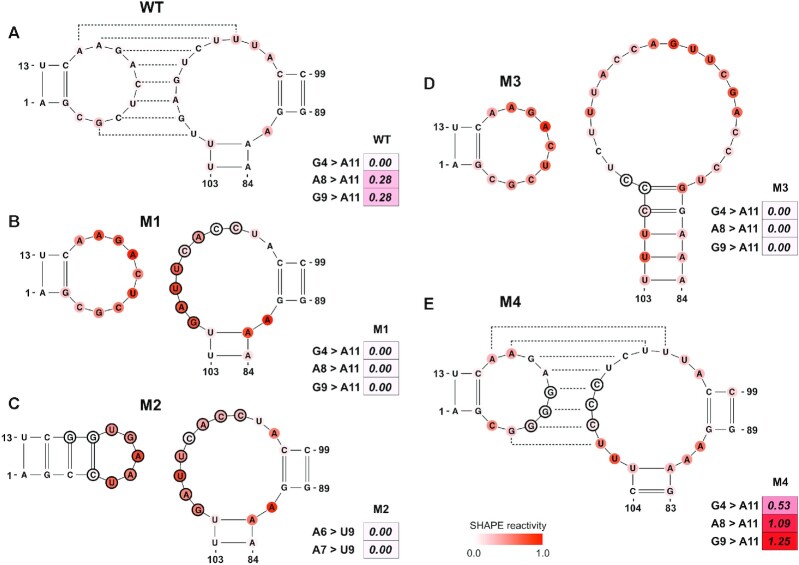
SHAPELoop identifies kissing loops in the *musD* transport element (MTE) and its mutants. Shown are loops and their SHAPE reactivities in wild-type MTE (**A**) and its mutants M1 (**B**), M2 (**C**), M3 (**D**) and M4 (**E**). Kissing loops in wild-type MTE and M4 mutant are shown as dashed lines. The SHAPE reactivities are shown on the structures using a coloring scheme, and penalties are shown as heat maps. Mutated residues are marked with black circles. The residues are renumbered compared to the original paper (72).

The first example is a study of the Murine *musD* transport element (MTE) (72), in which a nonaloop and a 1 × 10 nt internal loop form a kissing loop (Figure 5A). Since the low SHAPE reactivities provided a clue about the formation of a kissing loop, we intended to confirm this at a higher level of confidence using SHAPELoop penalties, similar to the other validation experiments conducted in that study. To this end, we assessed the nonaloop in the wild-type (WT) MTE. The SHAPE reactivities of two out of three residue pairs (A8 and A11, G9 and A11) moderately contradicted the characteristic SHAPE patterns (Figure 5A), suggesting that this hairpin loop may be involved in a higher-level architecture. We further validated this inference by examining the SHAPELoop penalties for the nonaloop in four MTE mutants: M1, M2, M3 and M4 (Figure 5B–E). The M1, M2, and M3 mutants were designed to disrupt the kissing loop architecture, and M4 was designed as an alternative form of kissing loop interaction. As expected, the penalties for M1, M2 and M3 were zero, whereas the penalty for M4 was 2.87. Accordingly, we can draw a convincing conclusion that the kissing loop formed in WT and M4 and was disrupted in M1, M2 and M3. In addition, the penalties suggest that the kissing loop interaction in M4 is stronger than that in WT, given that the penalty for M4 is higher than that for WT (2.87 versus 0.56). Unsurprisingly, these conclusions are in agreement with the results of the oligonucleotide hybridization experiments and biological function assays (72). Specifically, the biological functions of M1, M2 and M3 were severely impaired, whereas the function of M4 was enhanced compared to WT. Overall, these results indicate that SHAPELoop penalties can help researchers to test pseudoknots effectively and conveniently.

Similarly, we also examined SHAPELoop penalties for a tetraloop in the WT and mutant forms of *Peach latent mosaic viroid* (PLMVd.282) (73). Consistent with the conclusion of the original study, the penalty for the WT was higher than the penalty for the mutant (2.18 versus 0), supporting the conclusion that the kissing loop forms in the WT but not in the mutant (Supplementary Figure S10). Together, these results demonstrate that the characteristic SHAPE patterns identified by SHAPELoop are reliable, and the penalties calculated based on these patterns can be combined with mutagenesis experiments to credibly identify pseudoknots.

### SHAPELoop penalty is capable of capturing rearrangements of RNA secondary structures

**Figure 6.**
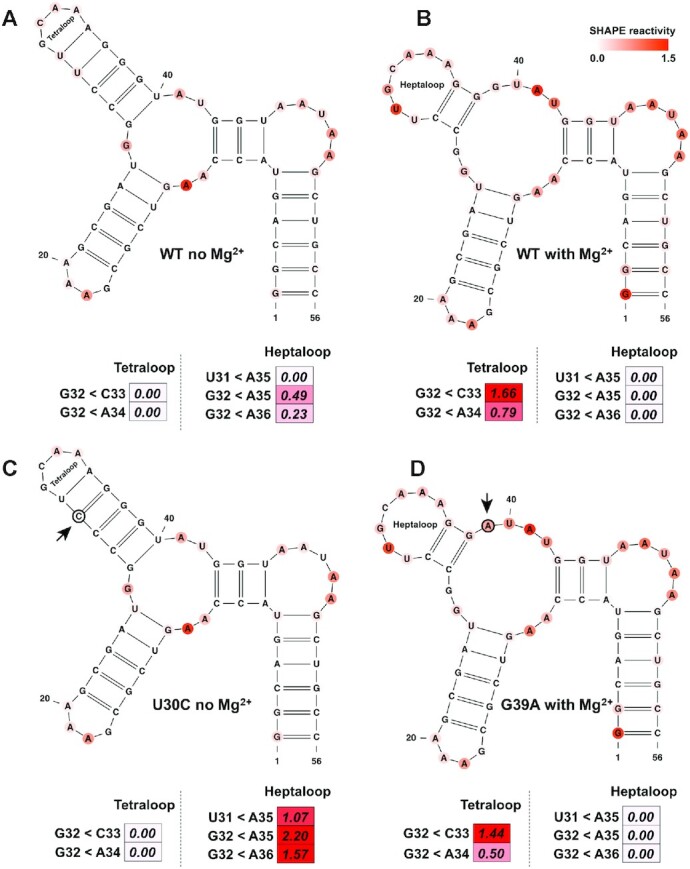
SHAPELoop accurately identifies the structural switch in the P5c subdomain. The structural switch between the tetraloop and the heptaloop is shown. The P5c subdomain folds into the tetraloop form in the absence of Mg^2+^ (**A**) and the heptaloop form in the presence of Mg^2+^ (**B**). (**C**) The U30C mutant, which is designed to stabilize the tetraloop. (**D**) The G39A mutant, which is designed to stabilize the heptaloop. The SHAPE reactivities are shown on the structures using a coloring scheme, and penalties are shown as heat maps. Mutated residues are marked with black circles. The residues are renumbered compared to the original paper (76).

Multivalent cations play an essential role in RNA structural stability (74). For instance, Mg^2+^ ions facilitate complex tertiary structures and folding arrangements that allow RNA molecules to perform various biological functions (75). Thus, we proceeded to investigate the ability of SHAPELoop penalties to recognize alternative RNA folding. To this end, we first evaluated the performance of SHAPELoop on the P5c subdomain of *Tetrahymena* *thermophila* group I intron ribozyme. The NMR and crystallography studies indicate that the P5c subdomain forms a tetraloop (Figure 6A) in the absence of Mg^2+^, and a heptaloop (Figure 6B) upon addition of Mg^2+^ (32,76,77). However, the RNAstructure-Fold algorithm predicted the same tetraloop structure when incorporating SHAPE data acquired in the absence or presence of Mg^2+^. We next examined the penalties for these two secondary structures to determine whether SHAPELoop could provide evidence of this Mg^2+^-induced secondary structural change. Figure 6A shows that, in the absence of Mg^2+^, the penalties for the tetraloop and the heptaloop were 0 and 0.72, respectively, indicating that the P5c subdomain folds into a tetraloop under this condition. On the contrary, once Mg^2+^ was added, the tetraloop penalty was higher than the heptaloop penalty (2.45 versus 0) (Figure 6B), suggesting the formation of a heptaloop in the presence of Mg^2+^. Additional evidence was gained from the penalties for the U30C mutant, which was designed to stabilize the tetraloop, and the G39A mutant, which was designed to stabilize the heptaloop. Figure 6C and D shows that the secondary structures with zero penalties were indeed the actual structures for the two mutants. Overall, these results provide valuable insights into the application of characteristic SHAPE patterns in identifying local structural changes in RNA.

**Figure 7.**
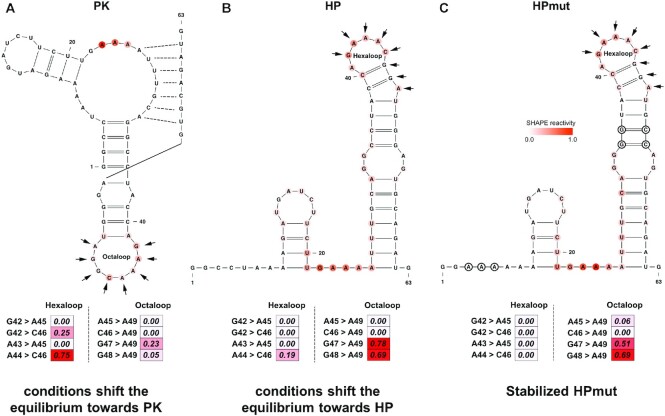
SHAPELoop identifies the structural switch in the 3′ splice site of influenza A segment 7 mRNA. Shown is the 63 nt structure in the 3′ splice site of influenza A segment 7 mRNA. This RNA switches between a pseudoknot structure (PK) (**A**) and a hairpin structure (HP) (**B**), with a predicted equilibrium constant close to 1. (**C**) The hairpin mutant (HPmut) folds completely into a hairpin structure. A splicing enhancer-binding site is marked with arrows. It folds into a distinct octaloop in PK and a distinct hexaloop in HP and HPmut. SHAPE reactivities are shown on structures using a coloring scheme, and penalties are shown as heat maps. Mutated residues are marked with black circles.

In addition to local structural switches, SHAPELoop recognizes rearrangements of global structures. Figure 7 shows an example of the structural rearrangement between a pseudoknot form and a hairpin-only form for a 63 nt RNA. This rearrangement was first discovered in the 3′ splice site of influenza A segment 8 mRNA by several NMR studies (78,79). Turner and colleagues later proposed that this structural change was also present in influenza A segment 7 mRNA (Figure 7A and B), which was further proven using a series of enzymatic (RNase T1, A and I_f_) and chemical (DMS, SHAPE, CMCT, DEPC and Pb^2+^) probing methods (80). Unfortunately, neither RNAstructure-Fold nor more specialized tools, such as ProbKnot (81) or ShapeKnots (82), could predict this structural switch. Therefore, in an attempt to decipher more structural information encoded in SHAPE data, we calculated SHAPELoop penalties for a distinct octaloop in the pseudoknot structure (PK) and a distinct hexaloop in the hairpin structure (HP). Figure 7A shows that, under a folding condition that shifted the equilibrium to the PK, the penalty for the octaloop was lower than that for the hexaloop (0.28 versus 1.00). Conversely, when another folding condition inverted the equilibrium to the HP, the penalty for the octaloop was higher than that for the hexaloop (1.47 versus 0.19) (Figure 7B). Of note, although the structure with the lower SHAPELoop penalty was the actual dominant structure for each folding condition, the penalty was not zero. This imperfect situation is reasonable because the predicted free energies of these two structures are similar (PK: −16.3 kcal/mol; HP: −16.9 kcal/mol) (80); thus, the predicted equilibrium constant is not far from 1, which means that the folding conditions may not push the equilibrium completely to one of these two structures. This explanation was proven by using an HP mutant (HPmut), to which specific mutations were introduced to inhibit pseudoknot folding (Figure 7C). For the HPmut, the penalty for the hexaloop was zero, whereas the penalty for the octaloop was 1.20. Taken together, the above results demonstrate that SHAPELoop is an effective and convenient tool for detecting local or global structural rearrangements under different folding conditions.

## DISCUSSION

In this work, we identified characteristic SHAPE patterns for a variety of loop motifs and developed a new method, SHAPELoop, to refine the structures predicted by existing thermodynamic folding models. Unlike models that consider only SHAPE-derived pseudo-energy for base pairing, SHAPELoop evaluates SHAPE patterns for loop motifs based on a posterior probabilistic model. Although RNAsc also evaluates residues in loop regions, it converts SHAPE reactivities into energy-tuning parameters, so the penalties imposed by loop regions can be easily overwhelmed by penalties from other regions (26). PatteRNA employs a GMM-HMM model to search for putative loop motifs based on given SHAPE data (36); however, this model considers only the influence between adjacent-neighbor residues, leading to an unsatisfactory prediction accuracy compared to SHAPELoop (Supplementary Figure S11).

SHAPELoop provides a novel insight into the interpretation of SHAPE probing data. In addition to improving the prediction accuracy of RNA secondary structures, it can also help to identify RNA secondary structural changes such as the local or global structural rearrangements, as demonstrated by several examples. The application of SHAPELoop to reference RNAs yielded the highest mean and median sensitivity and PPV values, compared to those generated using RNAstructure-Fold, RNAstructure-MaxExpect, RME, and SeqFold. However, the predictions of some RNAs exhibited little or no improvement. For these RNAs, the insufficient improvements are partially due to an inadequate representation of Boltzmann-weighted structure sampling (28,30). In other words, the candidate ensemble did not include the real structure. Alternatively, one can manually add structures of interest to the Boltzmann-weighted candidate ensemble, which may be useful when prior knowledge is available. Another major obstacle is the lack of characteristic SHAPE patterns for complex structural motifs, particularly for multi-branch loops (Supplementary Figure S9). These results underscore the necessity of identifying characteristic SHAPE patterns for more structural motifs.

SHAPELoop is moderately robust to noise in SHAPE data. Nonetheless, SHAPE experiments should be conducted with special care to maximize the data quality, for example, by strictly following standard protocols to avoid technical noise that may influence the outcome of structure modeling (10). Users can also integrate their benchmark datasets into SHAPELoop so that more SHAPE patterns will be reliably identified, especially for internal and multi-branch loops whose occurrences are rather scant in our benchmark RNA dataset. Accumulation of data may also help us understand why some loops deviate from the characteristic SHAPE patterns. We attempted to explain such deviations by comparing the conformations of tetraloops with the classic UUCG and GAAA loops (56,61) and found that the deviations could be partially due to experimental uncertainties; however, no further conclusions can be reached due to the paucity of data (Supplementary Figure S12).

The mechanism of SHAPE modification has attracted considerable interest, and several structure factors were shown to be linked to SHAPE reactivities (42,44–46,54). In this study, we examined the sugar conformations of loops extracted from non-redundant RNA structures from PDB and found that C2′-endo is overrepresented in residues with high SHAPE reactivities. Nevertheless, some characteristic SHAPE patterns are inconsistent with this sugar pucker preference, which might be caused by the incorrect assignment of the sugar pucker. Further investigations into SHAPE modifications will help us to better understand the identified SHAPE patterns.

High-throughput sequencing methods such as SHAPE-MaP (16,17) and icSHAPE (18) are widely used to measure the structures of whole transcriptomes, especially for in-cell analyses. The result of applying SHAPELoop to one SHAPE-MaP dataset (17) showed that this approach also improved prediction accuracy by integrating high-throughput SHAPE data (Supplementary Figure S13 and Supplementary Table S6). However, next-generation sequencing data are usually more vulnerable to uneven read coverage and noise arising from the complicated experimental procedure. Moreover, RNAs isolated from cells often bear various modifications such as m^6^A, which may induce local or global structural changes of RNAs (18,83) and affect the primer extension procedure in SHAPE experiments (10); therefore, users should select the optimal SHAPE experimental method according to their particular interests of the study.

## DATA AVAILABILITY

The source code of SHAPELoop is available at https://github.com/JingyiChris/SHAPELoop.

## Supplementary Material

gkab250_Supplemental_File
