# Supplementary material for: Characteristic chemical probing patterns of loop motifs improve prediction accuracy of RNA secondary structures
Source: Nucleic Acids Res. 2021 Apr 13;49(8):4294–307. doi: 10.1093/nar/gkab250 (PMC8096282; doi:10.1093/nar/gkab250)
Supplement: gkab250_Supplemental_File [file gkab250_supplemental_file.pdf]

Supplementary Information

Characteristic Chemical Probing Patterns of Loop Motifs Improve Prediction Accuracy of RNA Secondary Structures

Jingyi Cao and Yi Xue

School of Life Sciences, Tsinghua-Peking Joint Center for Life Sciences, Beijing Advanced Innovation Center for Structural Biology, Tsinghua University, Beijing, 100084, China

Table of Contents

SUPPLEMENTARY FIGURES......2

Supplementary Figure 1 | Comparison of SHAPE reactivities between two residues for each characteristic SHAPE pattern......2
Supplementary Figure 2 | Distribution of SHAPE difference for true and false loops......4
Supplementary Figure 3 | Distribution of SHAPE difference for each loop motif......5
Supplementary Figure 4 | Consistency between SHAPE patterns and sugar pucker preferences......6
Supplementary Figure 5 | Loop species and their populations in benchmark RNAs and RNAs deposited in PDB......7
Supplementary Figure 6 | The abundance of benchmark loops in PDB......8
Supplementary Figure 7 | SHAPELoop identifies loops that are falsely predicted by RNAstructure..9
Supplementary Figure 8 | SHAPELoop penalties for native, guidance, and SHAPELoop-predicted structures of *E. coli* 16S and 23S rRNAs......10
Supplementary Figure 9 | Prediction accuracies for multi-branch loops and non-multi-branch loops......11
Supplementary Figure 10 | SHAPELoop facilitates the identification of the kissing loop in *Peach latent mosaic viroid*......12
Supplementary Figure 11 | Comparison between SHAPELoop and patteRNA......13
Supplementary Figure 12 | Conformational comparison between loops matching and not matching the characteristic SHAPE patterns for the tetraloop motif......14
Supplementary Figure 13 | Comparison of SHAPE reactivities between two residues for each characteristic SHAPE pattern in the SHAPE-MaP dataset......15

SUPPLEMENTARY TABLES......16

Supplementary Table 1 | Details of the benchmark RNAs and the source of SHAPE data......16
Supplementary Table 2 | Details of the multi-branch loops extracted from benchmark RNAs......16
Supplementary Table 3 | Details of the characteristic SHAPE patterns for hairpin and bulge loop motifs......17
Supplementary Table 4 | Details of the characteristic SHAPE patterns for internal loop motifs......18
Supplementary Table 5 | Performances of SHAPELoop and other prediction tools on the CE SHAPE dataset......19
Supplementary Table 6 | Performances of SHAPELoop and other prediction tools on the SHAPE-MaP dataset......20

SUPPLEMENTARY REFERENCES......21

Supplementary Figures

Supplementary Figure 1 | Comparison of SHAPE reactivities between two residues for each characteristic SHAPE pattern

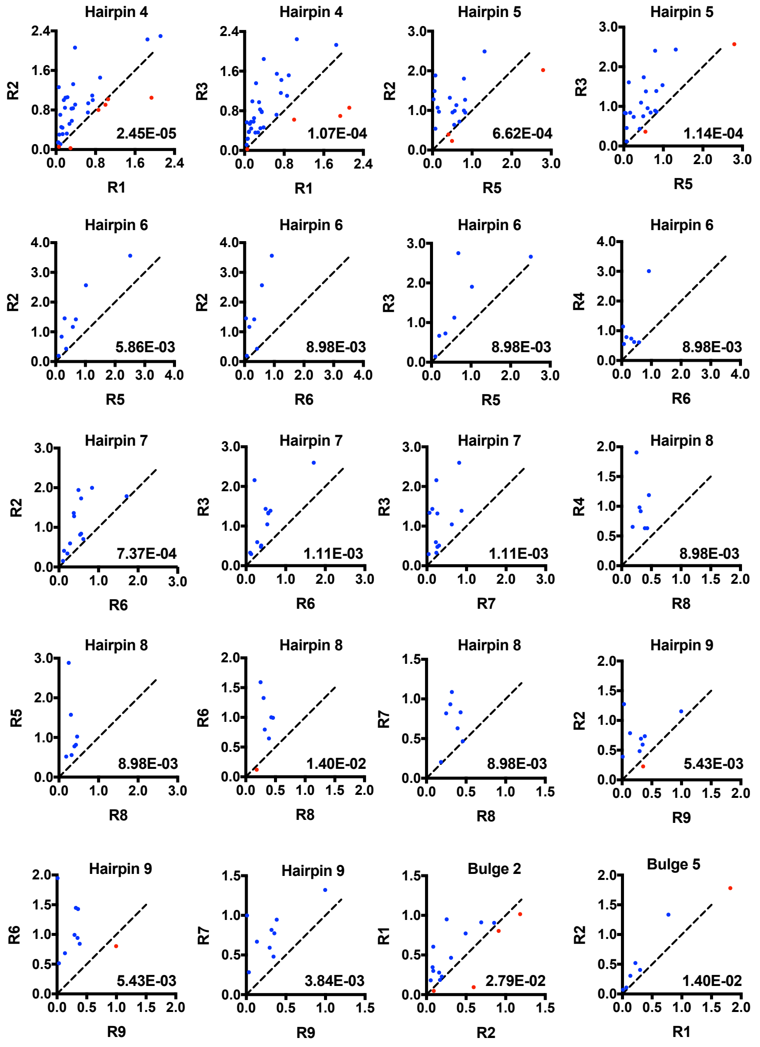

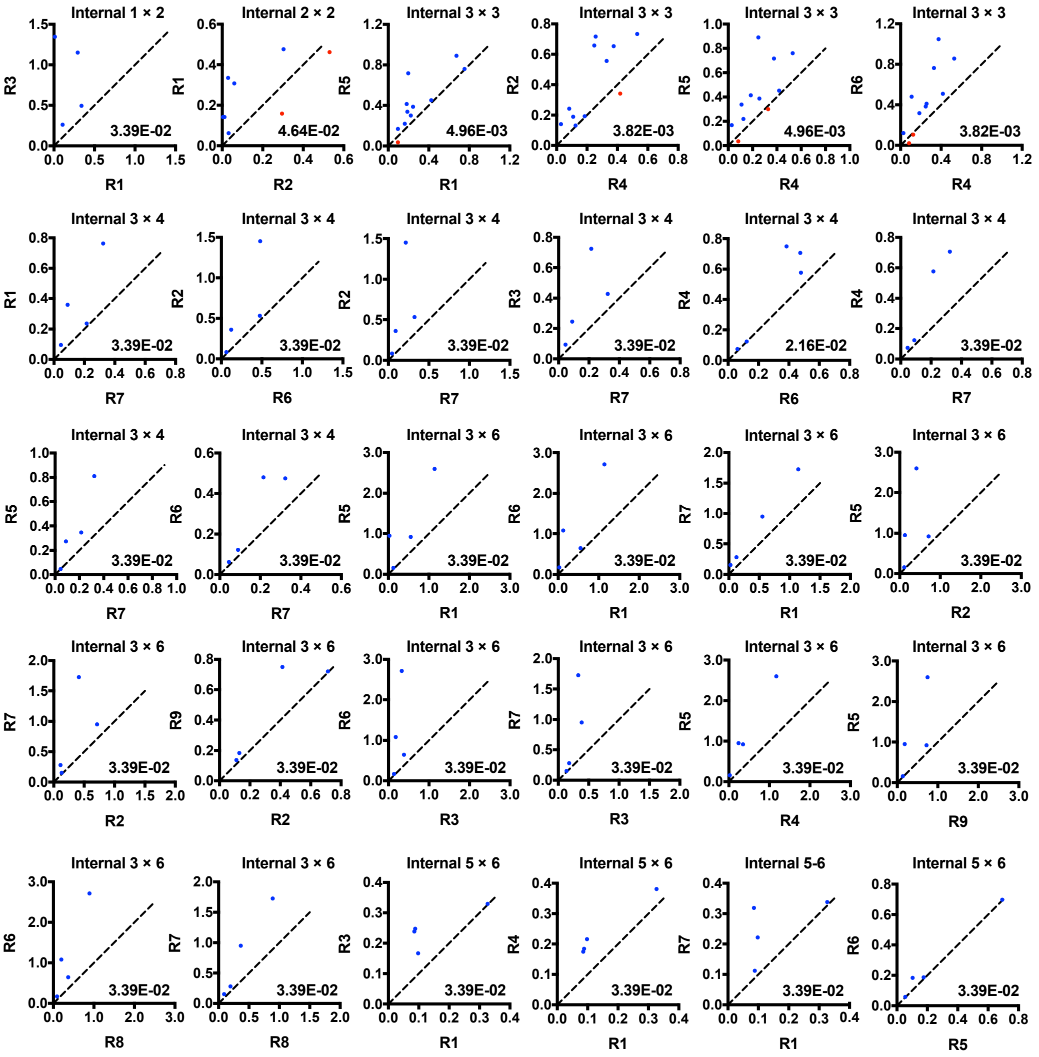

Scatter plots of the SHAPE reactivities of the two residues involved in each SHAPE pattern. Comparison pairs in disagreement with the characteristic SHAPE patterns are shown as red dots, while other pairs are shown as blue dots. The dashed lines indicate the same SHAPE reactivities between two residues. *P*-values were calculated using the paired Wilcoxon signed-rank test.

Supplementary Figure 2 | Distribution of SHAPE difference for true and false loops

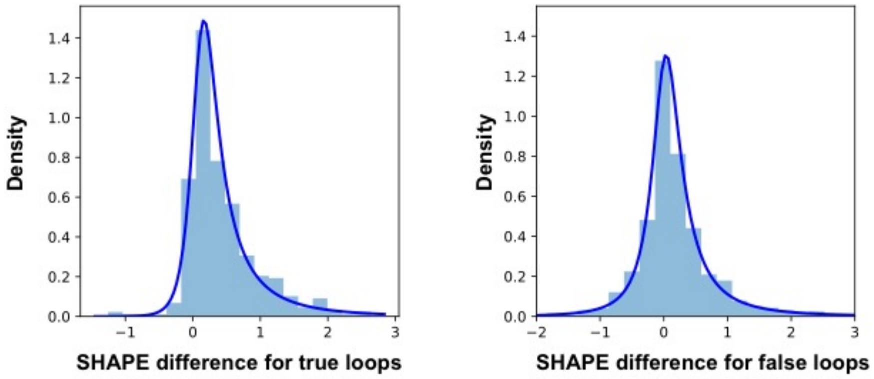

The distribution of SHAPE difference ($D_{i,j}$) between pairs of bases in true loops was fitted with a normal-inverse Gaussian distribution, while the distribution of $D_{i,j}$ for false loops was fitted with a Johnson's SU distribution. Both distributions passed the Kolmogorov–Smirnov test for goodness-of-fit. The parameters are: 0.753, 0.593, 0.100, 0.260 and -0.283, 0.825, 0.003, 0.246, respectively.

Supplementary Figure 3 | Distribution of SHAPE difference for each loop motif

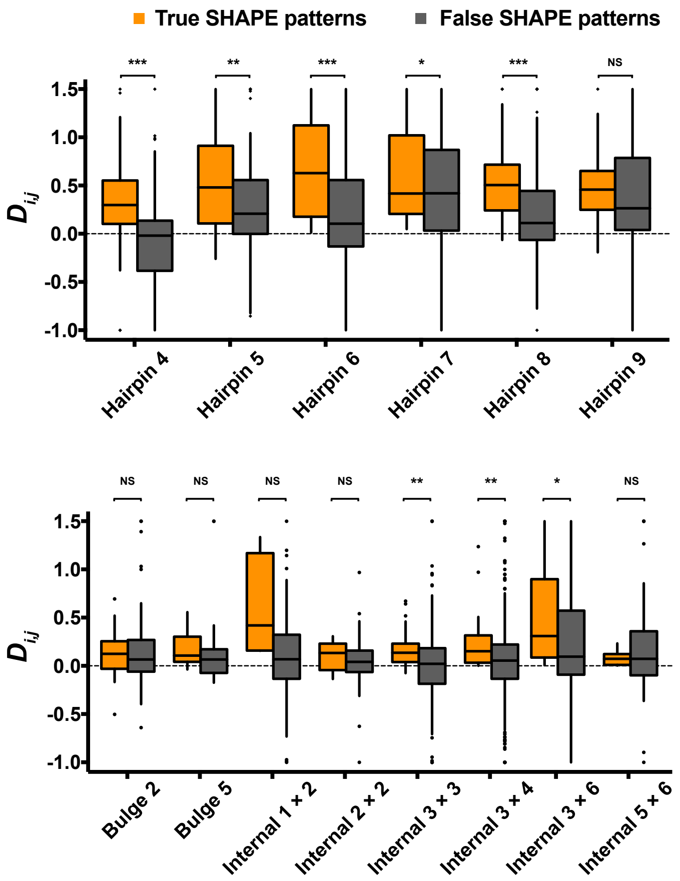

The SHAPE difference ($D_{i,j}$) for false SHAPE patterns arises from falsely-predicted loops extracted from the candidate ensemble of benchmark RNAs. The difference between true and false patterns was tested with the Student's t-test, and *P*-values are indicated by asterisks (*0.01 $<$ *P*-value $\leq$ 0.05; **0.001 $<$ *P*-value $\leq$ 0.01; ****P*-value $\leq$ 0.001).

Supplementary Figure 4 | Consistency between SHAPE patterns and sugar pucker preferences

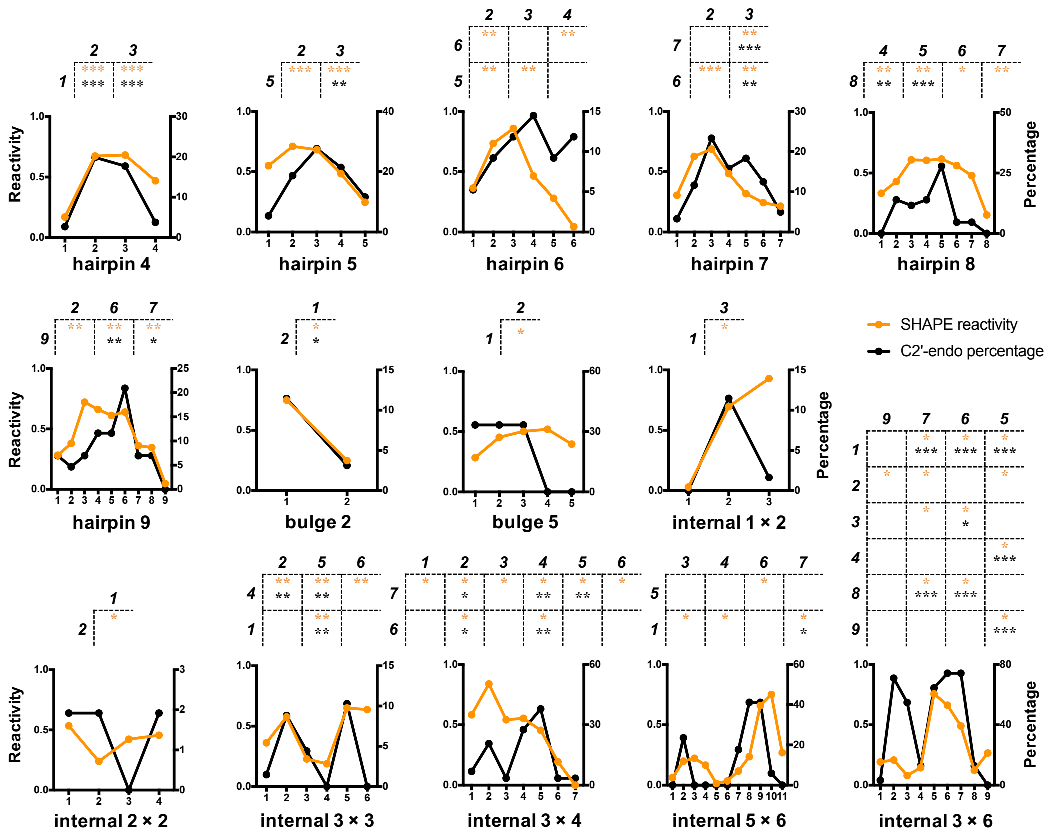

The averaged SHAPE reactivities at all positions of each loop motif in benchmark RNAs are shown by orange lines. The proportions of the C2′-endo sugar pucker conformation (for RNAs in the non-redundant PDB list with 3.0 Å cutoff) are shown by black lines. The characteristic SHAPE patterns of each loop motif are shown at the top of each subplot, with the residues at the top of each table showing higher SHAPE reactivities than the residues to the left, and their *P*-values are indicated by asterisks (*0.01 < *P*-value ≤ 0.05; **0.001 < *P*-value ≤ 0.01; ****P*-value ≤ 0.001). The residue position is counted from the 5′ end of each loop motif. Detailed *P*-values and sample sizes are listed in **Supplementary Tables 3–4**.

Supplementary Figure 5 | Loop species and their populations in benchmark RNAs and RNAs deposited in PDB

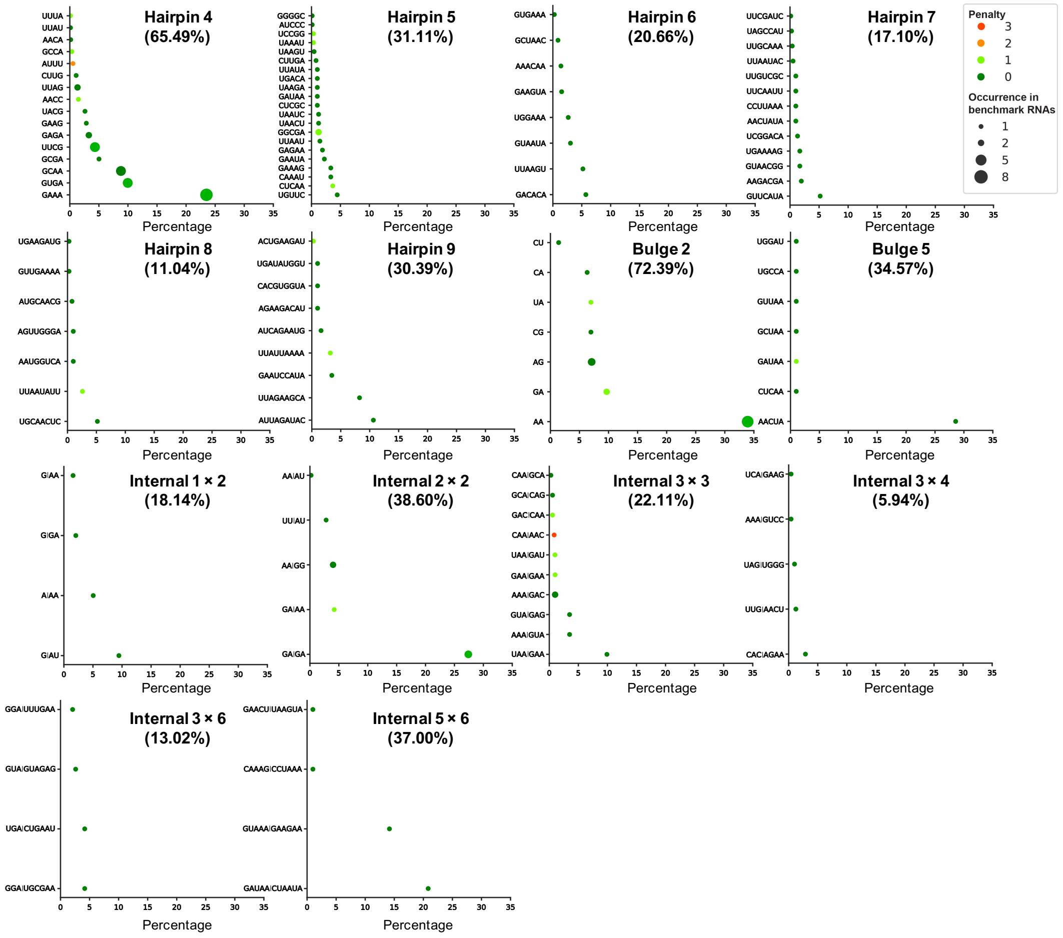

The number of each loop species in benchmark RNAs is shown as dots of different sizes, and the coloring scheme illustrates the average penalty. The x-axis represents the population of each loop species in the loop motif to which the loop species belongs in PDB. Numbers in brackets indicate the total population of that specific motif.

Supplementary Figure 6 | The abundance of benchmark loops in PDB

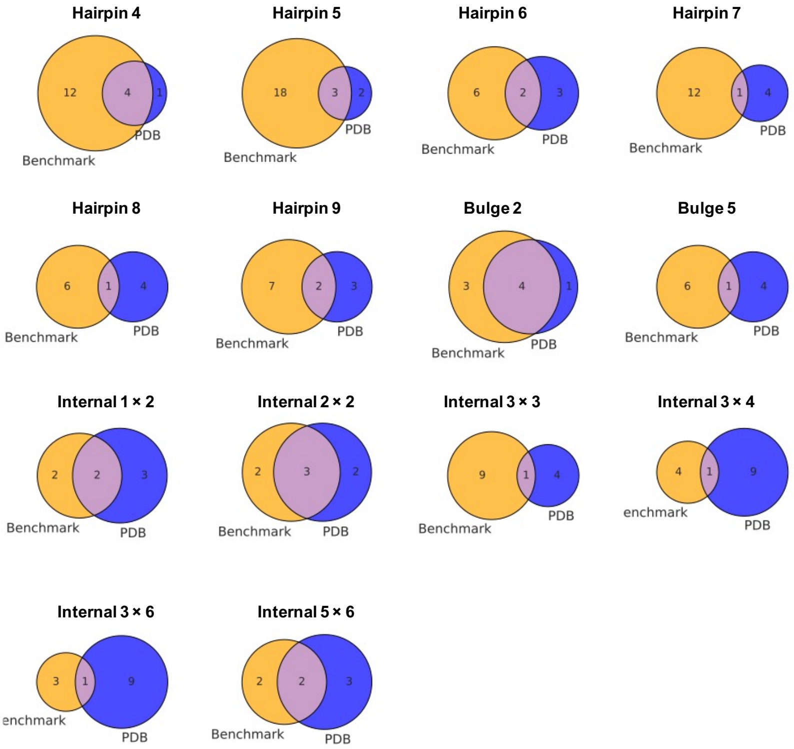

For each loop motif, shown is the number of overlapped loop species between loops in benchmark RNAs (benchmark loops) and the top five or top ten abundant loop species in PDB.

Supplementary Figure 7 | SHAPELoop identifies loops that are falsely predicted by RNAstructure

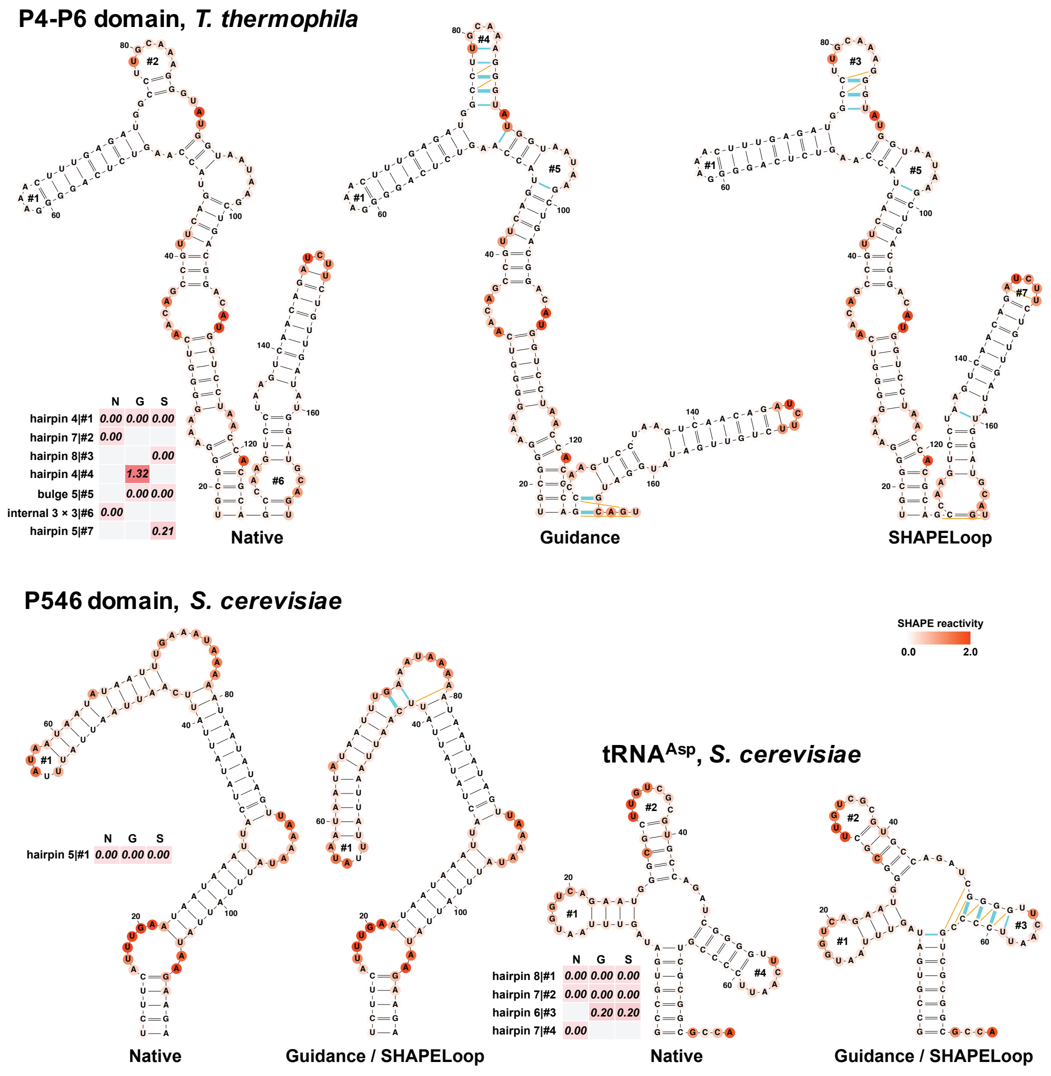

Shown are the native structures (N), RNAstructure-predicted guidance structures (G), and SHAPELoop-predicted structures (S) of three benchmark RNAs. SHAPE reactivities are shown on the structures using a coloring scheme. The penalties are shown as heat maps. The false-positive (incorrect) base pairs in the guidance or SHAPELoop-predicted structures are shown by green lines, and false-negative (missing) base pairs are shown by orange lines.

Supplementary Figure 8 | SHAPELoop penalties for native, guidance, and SHAPELoop-predicted structures of *E. coli* 16S and 23S rRNAs

16S rRNA domain 1

	G	N	S
bulge 2|64		1.91	
internal 2 × 2|65			0.00
internal 4 × 3|65	3.05		
internal 1 × 2|68		0.00	
bulge 2|71	0.00	0.00	
hairpin 4|83	0.00	0.00	0.00
internal 2 × 1|129	0.00	0.00	0.00
internal 5 × 6|148		0.00	
hairpin 4|159	0.00	0.00	0.00
hairpin 4|187	0.00	0.00	0.00
bulge 2|204		0.79	
hairpin 4|208		1.13	
bulge 2|250	0.00	0.00	0.00
hairpin 7|260	0.00	0.00	0.00
hairpin 9|296	0.68		0.68
hairpin 4|297		0.00	
hairpin 7|324			0.00
hairpin 5|325	1.01		
hairpin 4|343	0.00	0.00	0.00
hairpin 4|380	0.00	0.00	0.00
internal 6 × 5|410	0.08	0.08	0.08
hairpin 4|420	0.00	0.00	0.00
hairpin 7|463	0.00	0.00	0.00
bulge 5|516		0.00	
hairpin 4|523	0.77		0.77

16S rRNA domain 2

	G	N	S
internal 3 × 3|20	0.09	0.09	0.09
internal 1 × 2|35	0.26		0.26
hairpin 5|57	0.87	0.87	0.87
bulge 2|80		0.32	
internal 2 × 2|102	1.11		1.11
internal 3 × 3|113	0.46	0.46	0.46
internal 3 × 4|124	4.16		
internal 3 × 6|124		0.09	0.09
hairpin 6|130		0.06	0.06
hairpin 8|130	0.00		
hairpin 4|166	0.00	0.00	0.00
internal 2 × 2|215	0.95		
internal 3 × 3|219		0.00	
hairpin 9|226	0.00	0.00	0.00
hairpin 5|280	0.00	0.00	0.00
hairpin 4|302	1.90		
bulge 5|308		0.00	
hairpin 4|337	0.00	0.00	0.00

16S rRNA domain 3

	G	N	S
bulge 2|42	0.00		0.00
hairpin 8|48	0.00	0.00	
hairpin 4|97	0.00	0.00	0.00
hairpin 4|113	0.00	0.00	0.00
bulge 2|138		0.00	
internal 1 × 2|138	1.08		
hairpin 4|161		0.29	
hairpin 6|174	0.00	0.00	0.00
internal 3 × 4|209	0.00	0.00	0.00
bulge 2|214	0.00	0.00	0.00
hairpin 5|219		1.10	
hairpin 5|250	0.00	0.00	0.00
internal 3 × 3|344	0.31	0.31	0.31
hairpin 4|350	0.00	0.00	0.00
hairpin 8|399	0.00	0.00	0.00
hairpin 9|441	0.00	0.00	0.00

16S rRNA domain 4

	G	N	S
internal 3 × 6|5	0.00		0.00
internal 3 × 4|10	0.00		0.00
internal 2 × 2|17	0.00		0.00
internal 2 × 2|21	0.00	0.00	0.00
internal 4 × 3|35		0.00	
internal 2 × 1|50		0.00	
internal 2 × 2|50	0.00		0.00
hairpin 4|54		0.00	
hairpin 4|120	0.00	0.00	0.00
hairpin 5|136	0.00		0.00

G: Guidance

N: Native

S: SHAPELoop

23S rRNA domain 1

	G	N	S
bulge 5|25		0.00	
bulge 2|49			0.05
internal 2 × 1|51	1.59		
hairpin 6|55	0.00		0.00
bulge 2|63			0.43
internal 2 × 2|83		0.00	
hairpin 7|91	0.00		0.00
hairpin 6|117	0.00		
hairpin 4|124		0.25	0.25
hairpin 4|138	0.00	0.00	0.00
hairpin 9|159	0.00	0.00	0.00
hairpin 4|179	3.78		
internal 2 × 1|189			0.00
internal 5 × 6|189		0.30	
hairpin 4|196		0.00	
bulge 2|240	0.25		0.25
internal 1 × 2|244	0.00		0.00
internal 6 × 3|275		0.00	
hairpin 6|306		0.00	
hairpin 4|307	0.36		0.36
hairpin 5|328		0.00	
hairpin 6|328			0.37
hairpin 4|329	13.9		
hairpin 9|383		0.00	
hairpin 4|412	0.00		0.00
hairpin 4|463	0.00	0.00	0.00
hairpin 5|488	0.00	0.00	0.00
internal 2 × 2|537	0.00	0.00	0.00

23S rRNA domain 2

	G	N	S
internal 2 × 2|46	0.00	0.00	0.00
hairpin 5|50	0.00	0.00	0.00
hairpin 4|68	0.04	0.04	
hairpin 5|80	0.00	0.00	0.00
hairpin 5|113			0.00
internal 3 × 4|142	0.00	0.00	0.00
hairpin 6|151	0.00	0.00	0.00
hairpin 8|183	0.00	0.00	0.00
hairpin 5|218		0.00	0.00
hairpin 7|237	0.00		0.00
hairpin 4|265	0.00	0.00	0.00
internal 3 × 3|283	2.04		
internal 3 × 3|314	1.12	1.12	1.12
hairpin 5|324	0.00	0.00	0.00
hairpin 8|393	0.07		0.07
hairpin 6|394		0.00	
hairpin 6|417		0.00	
internal 3 × 3|437		0.00	
internal 2 × 2|438	0.00		0.00
internal 2 × 1|470		0.00	
internal 2 × 2|477		0.00	
bulge 2|483	0.00		0.00
internal 2 × 2|498		0.00	
hairpin 9|503		0.00	
hairpin 7|504	0.00		0.00
hairpin 6|531	0.00	0.00	0.00
bulge 5|577		0.00	
hairpin 4|611	0.00	0.00	0.00
internal 6 × 3|647		0.07	
hairpin 4|661	0.00	0.00	0.00

23S rRNA domain 3

	G	N	S
hairpin 7|12		0.00	
hairpin 5|13	0.00		0.00
bulge 2|51	0.00		0.00
hairpin 5|55	0.00	0.00	0.00
internal 3 × 3|82	0.25	0.25	0.25
internal 3 × 3|88	0.00		0.00
hairpin 4|94	0.00	0.00	0.00
hairpin 8|120	0.44	0.44	
internal 2 × 2|148	0.69	0.69	0.69
hairpin 7|182	0.20		0.20
internal 3 × 3|198	1.27		1.27
internal 1 × 2|205			0.00
internal 3 × 3|205	0.00	0.00	
hairpin 5|223	0.00	0.00	0.00
bulge 2|238	0.00	0.00	
hairpin 4|264	0.00	0.00	0.00
hairpin 6|343	0.00	0.00	0.00
hairpin 5|361	0.00	0.00	0.00

23S rRNA domain 4

	G	N	S
hairpin 6|31	0.00		
internal 3 × 3|75	0.39		
hairpin 5|82	0.00	0.00	0.00
internal 3 × 4|124	3.02		
hairpin 9|132		0.88	
internal 3 × 3|155		0.09	
hairpin 4|161	0.00	0.00	0.00
bulge 2|192		0.00	
bulge 2|201	0.00	0.00	0.00
bulge 2|218	0.00		0.00
hairpin 4|223		0.00	
hairpin 9|223	0.00		0.00
bulge 2|253		0.00	
hairpin 7|266		0.00	
hairpin 5|305	0.00	0.00	0.00
hairpin 6|319			0.00
hairpin 9|329	0.00		

23S rRNA domain 5

	G	N	S
hairpin 8|13		0.09	
hairpin 6|14	0.00		0.00
internal 3 × 4|53			0.00
internal 3 × 4|66		0.03	
internal 1 × 2|67	3.64		3.64
bulge 5|94	0.00		
internal 2 × 2|110			0.00
internal 1 × 2|111	0.00		
internal 6 × 3|115		0.04	
hairpin 4|129	0.15	0.15	0.15
hairpin 5|195	0.00	0.00	0.00
hairpin 5|235		0.00	
hairpin 7|290		0.00	
hairpin 5|291	0.00		0.00
hairpin 4|312	3.16		3.16
internal 2 × 2|336	0.00		0.00
hairpin 6|341			0.00
hairpin 4|342	4.01		
hairpin 7|390	0.00		0.00
bulge 2|396		0.00	
bulge 5|407		0.00	
bulge 5|414		0.29	
bulge 2|424		0.00	
internal 3 × 3|452	0.00	0.00	0.00
hairpin 7|457	0.00	0.00	0.00
hairpin 6|514	0.00	0.00	0.00
hairpin 5|537	0.00	0.00	0.00
hairpin 4|580	0.00	0.00	0.00

23S rRNA domain 6

	G	N	S
internal 2 × 2|13		0.42	
internal 2 × 1|28	0.03		0.03
hairpin 8|32	0.15		0.15
internal 2 × 2|63	0.00		0.00
bulge 2|87	0.00	0.00	0.00
hairpin 7|195		0.00	0.00
hairpin 4|232	0.00	0.00	0.00
hairpin 5|271	0.00		0.00

Shown are penalties for the native structures (N), RNAstructure-predicted guidance structures (G), and SHAPELoop-predicted structures (S) of *E. coli* 16S and 23S rRNAs. Loops not present are shown as blank.

Supplementary Figure 9 | Prediction accuracies for multi-branch loops and non-multi-branch loops

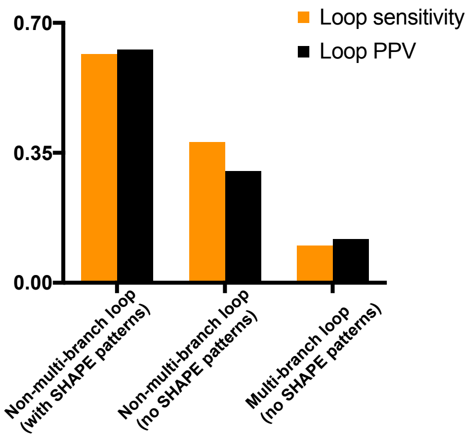

Loops in reference and SHAPELoop-predicted structures are divided into three categories as follows: non-multi-branch loops whose characteristic SHAPE patterns were identified, non-multi-branch loops without characteristic SHAPE patterns, and multi-branch loops (also without characteristic SHAPE patterns). The sensitivity and PPV were calculated at the level of loops instead of base pairs.

Supplementary Figure 10 | SHAPELoop facilitates the identification of the kissing loop in *Peach latent mosaic viroid*

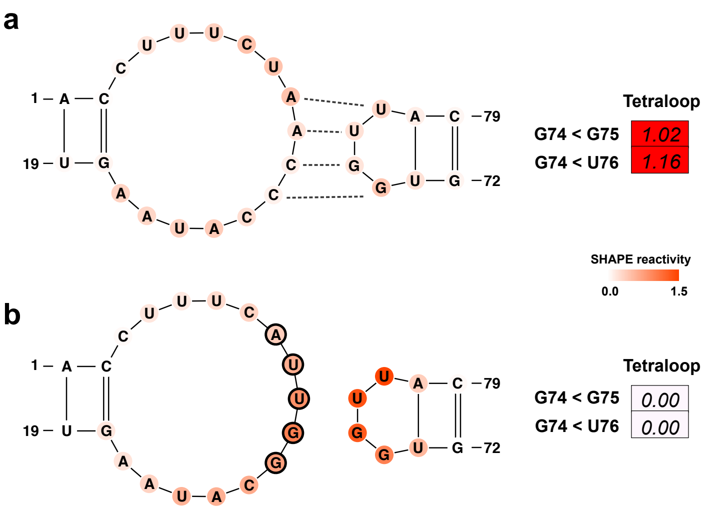

A kissing loop (indicated by dashed lines) forms in the wild type *Peach latent mosaic viroid* **(a)** and is disrupted in the mutant **(b)**. The SHAPE reactivities are shown on the structures using a coloring scheme, and penalties are shown as heat maps. Mutated residues are marked with black circles. The residues are renumbered compared to the original paper (1).

Supplementary Figure 11 | Comparison between SHAPELoop and patteRNA

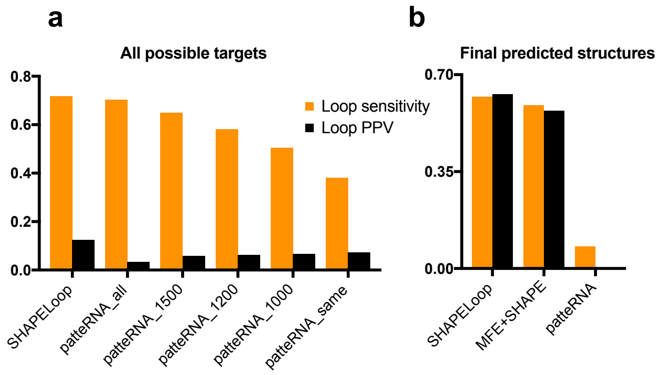

c

	Accuracy
SHAPELoop	0.87
RNAstructure + SHAPE	0.85
RNAstructure	0.80
patteRNA - Viterbi	0.71
patteRNA - posteriors	0.70

(a) Performances of patteRNA and SHAPELoop in searching for all possible loop targets. When evaluating all possible loops identified by patteRNA, the results were filtered with the following criteria: all loops with positive scores, resulting in 2748 loops; the top N loops with the highest scores, where N is 1500, 1200, 1000, and 688 (i.e., the number of SHAPELoop-identified loops from candidate ensemble). **(b)** Prediction accuracies for loops in patteRNA or SHAPELoop-predicted structures. The loop PPV is not available for patteRNA-predicted structures because those structures are sequences of nucleotide pairing states, and no information about pairing partners can be inferred. **(c)** Performances of patteRNA, SHAPELoop, and other tools in predicting pairing states. The accuracy was calculated with the $\ell 1$-norm distance that was also used in the patteRNA paper.

Supplementary Figure 12 | Conformational comparison between loops matching and not matching the characteristic SHAPE patterns for the tetraloop motif

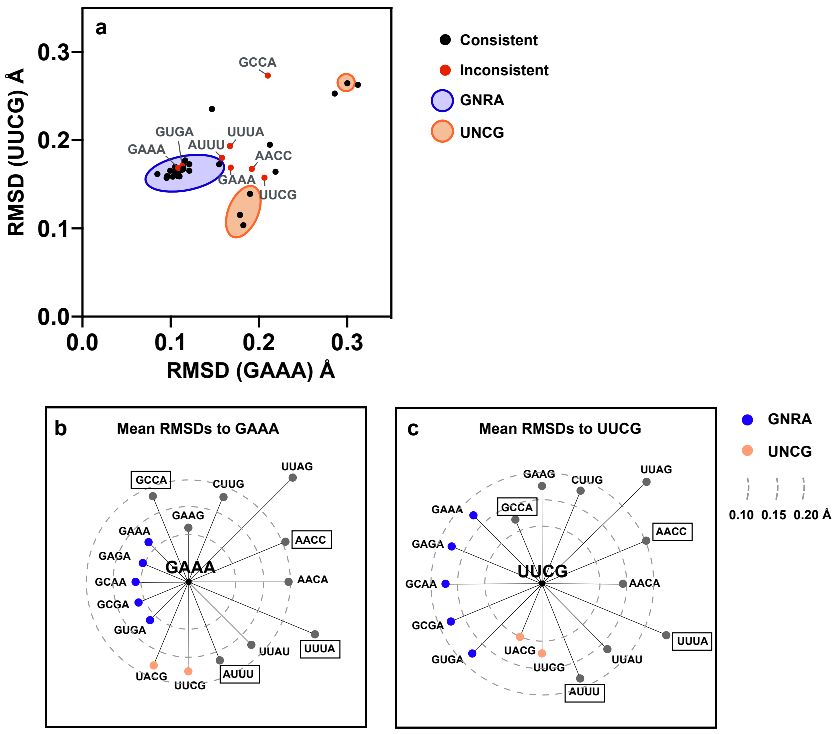

(a) The root-mean-square deviations (RMSDs) between each of 34 tetraloops (corresponding to 16 non-redundant tetraloops) in benchmark RNAs and the classic GAAA (2) or UUCG (3) loop. RMSDs were calculated after alignment according to backbone atoms (P, C3′, C4′, C5′, O3′, and O5′). The coordinates of 34 tetraloops were extracted from the PDB structures associated with the benchmark RNAs (see **Supplementary Table 1**). The PDB accession codes of the classic GAAA and UUCG structures are 1ZIF and 1HLX, respectively. GNRA (N = any, R = A/G) loops matching the characteristic SHAPE patterns are surrounded by a blue circle, while UNCG loops by two orange circles. Eight loops whose reactivities are inconsistent with the characteristic SHAPE patterns are shown as red dots, otherwise they are shown as black dots. Among these eight loops in red, two are located inside the GNRA cluster, suggesting the SHAPE data of some loops likely deteriorate due to experimental noise. It is worth noting that there are eight GAAA in total, and six of them are consistent with SHAPE patterns. **(b and c)** The mean RMSDs between these 16 non-redundant loops and the classic GAAA or UUCG by averaging RMSDs for loops in non-redundant RNAs deposited in PDB. The loop types that do not match SHAPE patterns are indicated by black boxes. Although these unmatched loop types in **(b)** (i.e., GCCA, AACC, UUUA, and AUUU) indeed show relatively large RMSDs, the same phenomenon cannot be observed in **(c)**. Besides, each of these unmatched loops contains only one member, making it difficult to draw any statistically meaningful conclusion.

Supplementary Figure 13 | Comparison of SHAPE reactivities between two residues for each characteristic SHAPE pattern in the SHAPE-MaP dataset

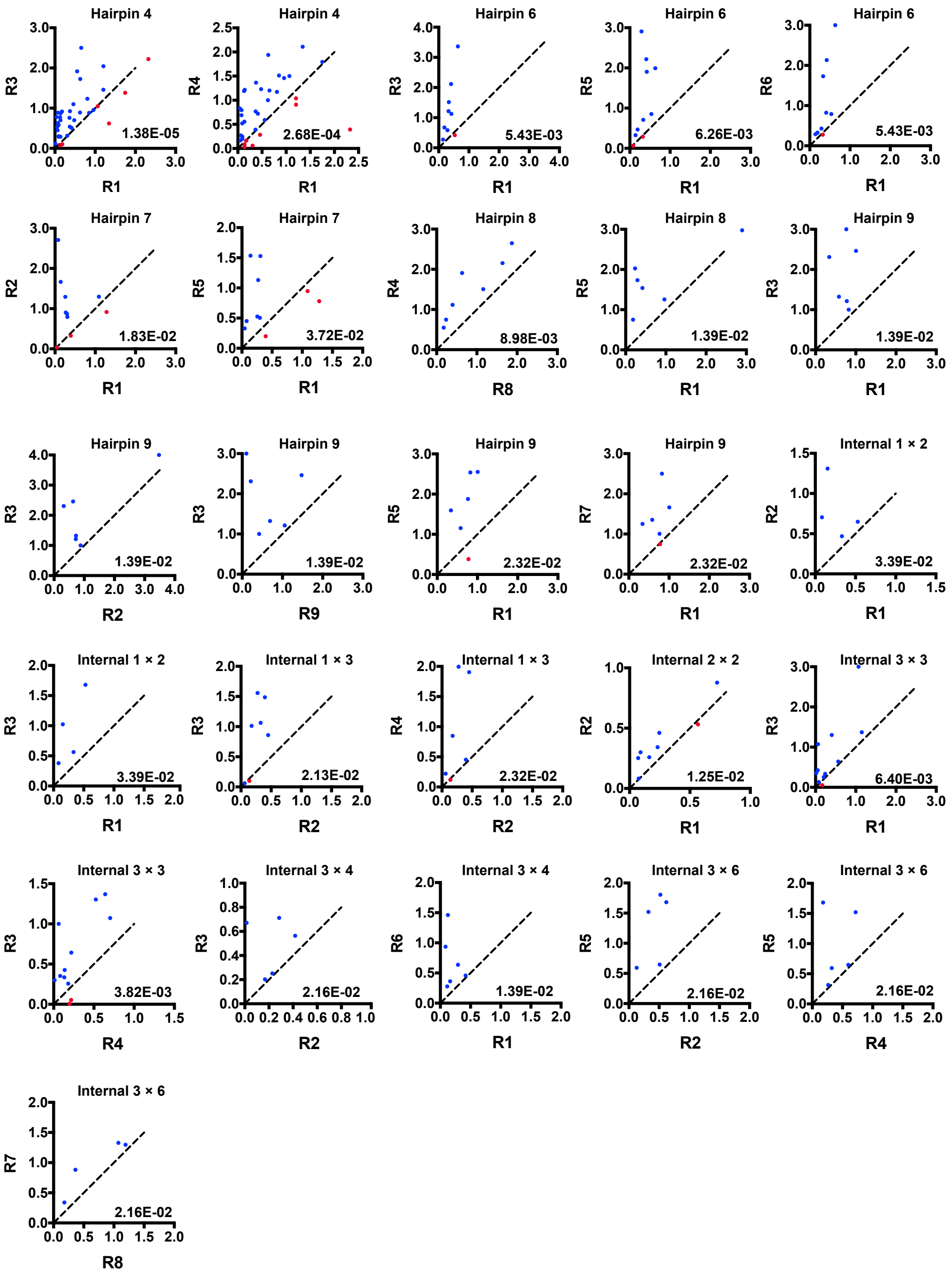

Scatter plots of the SHAPE reactivities of the two residues involved in each SHAPE pattern. Comparison pairs in disagreement with the characteristic SHAPE patterns are shown as red dots, while other pairs are shown as blue dots. The dashed lines indicate the same SHAPE reactivities between two residues. *P*-values were calculated using the paired Wilcoxon signed-rank test.

Supplementary Tables

Supplementary Table 1 | Details of the benchmark RNAs and the source of SHAPE data

**RNA**	**Species**	**Length**	**Reference of known structure[1]**	**Reference of SHAPE data[1]**
16S rRNA	*E. coli*	1542	(4)	(4)
23S rRNA	*E. coli*	2904	(4)	(4)
5S rRNA	*E. coli*	170	(5)	(5)
adenine riboswitch	*V. vulnificus*	121	(5)	(5)
cyclic di-GMP riboswitch	*V. cholerae*	135	(5)	(5)
glycine riboswitch	*F. nucleatum*	198	(5)	(5)
P546 domain, group I intron	*S. cerevisiae*	155	(6)	(7)
$tRNA^{Asp}$	*S. cerevisiae*	75	(8)	(9)
$tRNA^{phe}$	*E. coli*	116	(5)	(5)
P4-P6 domain of group I intron	*T. thermophila*	202	(5)	(5)
domain II of the HCV IRES[2]	*Hepatitis C virus*	95	(10)	(4)

[1]: The source of the data (see Supplementary References).
[2]: Internal ribosome entry sequence.

Supplementary Table 2 | Details of the multi-branch loops extracted from benchmark RNAs

	**Total number**	**Motif number**	**Length range**
3-branch loop	38	35	1–22
4-branch loop	12	12	2–18
5-branch loop	9	9	3–27
7-branch loop	1	1	19

Supplementary Table 3 | Details of the characteristic SHAPE patterns for hairpin and bulge loop motifs

Loop Type	Length	SHAPE					C2´ -endo		
		SHAPE patterns[1]	Sample size[2]	Matched ratio[3]	*P*-value[4]	Adjusted *P*-value[5]	Sample size[6]	*P*-value[7]	*P*-value[8]
Hairpin	4	2 > 1	34	0.82	2.45E-05	3.33E-04	811	5.92E-27	1.01E-14
		3 > 1	34	0.88	1.07E-04	4.42E-04	811	5.34E-26	2.42E-13
Hairpin	5	2 > 5	20	0.85	6.62E-04	3.31E-03	237	1.95E-02	5.12E-02
		3 > 5	22	0.91	1.14E-04	1.14E-03	237	7.44E-06	2.21E-03
Hairpin	6	2 > 5	8	1.00	5.86E-03	3.37E-02	197	7.93E-01	5.00E-01
		2 > 6	7	1.00	8.98E-03	3.37E-02	197	8.23E-01	7.18E-01
		3 > 5	7	1.00	8.98E-03	3.37E-02	197	2.33E-01	2.40E-01
		4 > 6	7	1.00	8.98E-03	3.37E-02	197	4.32E-02	2.96E-01
Hairpin	7	2 > 6	13	1.00	7.37E-04	4.55E-02	271	8.23E-01	5.91E-01
		3 > 6	12	1.00	1.11E-03	4.55E-02	271	6.57E-03	6.18E-03
		3 > 7	12	1.00	1.11E-03	4.55E-02	271	2.66E-05	5.03E-05
Hairpin	8	4 > 8	7	1.00	8.98E-03	8.38E-02	104	1.56E-04	7.15E-03
		5 > 8	7	1.00	8.98E-03	8.38E-02	104	1.36E-06	2.66E-04
		6 > 8	7	0.86	1.40E-02	9.80E-02	104	2.34E-03	7.86E-02
		7 > 8	7	1.00	8.98E-03	8.38E-02	104	2.34E-03	7.86E-02
Hairpin	9	2 > 9	9	0.89	5.43E-03	4.45E-02	115	7.15E-03	7.86E-02
		6 > 9	9	0.89	5.43E-03	4.45E-02	115	2.87E-07	1.35E-03
		7 > 9	9	1.00	3.84E-03	4.45E-02	115	2.66E-04	4.16E-02
Bulge	2	1 > 2	16	0.75	2.79E-02	2.79E-02	195	4.82E-05	1.05E-02
Bulge	5	2 > 1	7	0.86	1.40E-02	1.40E-01	6	1.59E-01	NA

[1]: The residue position is counted from the 5´ end of each loop motif.
[2]: The number of loops in reference RNAs (loops without SHAPE reactivities at the positions involved in that SHAPE pattern were filtered out).
[3]: The proportion of loops that match the SHAPE pattern.
[4]: Wilcoxon signed-rank test for SHAPE reactivities between positions involved in each SHAPE pattern.
[5]: *P*-values that were corrected with the Benjamini–Hochberg multiple test.
[6]: The number of loops in non-redundant RNAs deposited in PDB.
[7]: Wilcoxon signed-rank test for C2´-endo populations between positions involved in each SHAPE pattern (resolution cutoff 4.0 Å).
[8]: Wilcoxon signed-rank test for C2´-endo populations between positions involved in each SHAPE pattern (resolution cutoff 3.0 Å).

Supplementary Table 4 | Details of the characteristic SHAPE patterns for internal loop motifs

Loop type	Length	SHAPE					C2′-endo		
		SHAPE patterns[1]	Sample size[2]	Matched ratio[3]	*P*-value[4]	Adjusted *P*-value[5]	Sample size[6]	*P*-value[7]	*P*-value[8]
Internal	1 × 2	3 > 1	4	1.00	3.39E-02	1.02E-01	168	5.12E-02	1.59E-01
Internal	2 × 2	1 > 2	8	0.75	4.64E-02	2.79E-01	145	2.82E-01	5.00E-01
Internal	3 × 3	5 > 1	11	0.91	4.96E-03	2.48E-02	164	3.76E-03	7.15E-03
		2 > 4	11	0.91	3.82E-03	2.48E-02	164	1.95E-03	7.15E-03
		5 > 4	11	0.82	4.96E-03	2.48E-02	164	6.70E-04	4.08E-03
		6 > 4	11	0.82	3.82E-03	2.48E-02	164	8.41E-01	NA
Internal	3 × 4	1 > 7	4	1.00	3.39E-02	8.91E-02	54	1.59E-01	2.82E-01
		2 > 6	4	1.00	3.39E-02	8.91E-02	54	6.28E-03	2.94E-02
		2 > 7	4	1.00	3.39E-02	8.91E-02	54	1.95E-03	2.94E-02
		3 > 7	4	1.00	3.39E-02	8.91E-02	54	2.82E-01	5.00E-01
		4 > 6	5	1.00	2.16E-02	8.91E-02	54	9.82E-03	4.08E-03
		4 > 7	4	1.00	3.39E-02	8.91E-02	54	5.71E-03	9.82E-03
		5 > 7	4	1.00	3.39E-02	8.91E-02	54	1.00E-05	1.95E-03
		6 > 7	4	1.00	3.39E-02	8.91E-02	54	2.82E-01	5.00E-01
Internal	3 × 6	5 > 1	4	1.00	3.39E-02	1.36E-01	54	7.28E-09	1.69E-05
		6 > 1	4	1.00	3.39E-02	1.36E-01	54	7.28E-09	3.55E-06
		7 > 1	4	1.00	3.39E-02	1.36E-01	54	1.56E-09	3.55E-06
		5 > 2	4	1.00	3.39E-02	1.36E-01	54	5.00E-01	7.36E-01
		7 > 2	4	1.00	3.39E-02	1.36E-01	54	2.74E-01	3.91E-01
		9 > 2	4	1.00	3.39E-02	1.36E-01	54	1.00E+00	1.00E+00
		6 > 3	4	1.00	3.39E-02	1.36E-01	54	3.09E-01	1.69E-02
		7 > 3	4	1.00	3.39E-02	1.36E-01	54	1.38E-01	6.68E-02
		5 > 4	4	1.00	3.39E-02	1.36E-01	54	8.03E-08	1.73E-04
		5 > 9	4	1.00	3.39E-02	1.36E-01	54	1.65E-09	3.87E-06
		6 > 8	4	1.00	3.39E-02	1.36E-01	54	2.16E-08	6.54E-06
		7 > 8	4	1.00	3.39E-02	1.36E-01	54	2.42E-07	1.28E-04
Internal	5 × 6	3 > 1	4	1.00	3.39E-02	4.67E-01	40	7.18E-01	NA
		4 > 1	4	1.00	3.39E-02	4.67E-01	40	7.18E-01	NA
		7 > 1	4	1.00	3.39E-02	4.67E-01	40	4.16E-02	4.16E-02
		6 > 5	4	1.00	3.39E-02	4.67E-01	40	8.41E-01	NA

[1]: The residue position is counted from the 5′ end of each loop motif.
[2]: The number of loops in reference RNAs (loops without SHAPE reactivities at the positions involved in that SHAPE pattern were filtered out).
[3]: The proportion of loops that match the SHAPE pattern.
[4]: Wilcoxon signed-rank test for SHAPE reactivities between positions involved in each SHAPE pattern.
[5]: *P*-values that were corrected with the Benjamini–Hochberg multiple test.
[6]: The number of loops in non-redundant RNAs deposited in PDB.
[7]: Wilcoxon signed-rank test for C2′-endo populations between positions involved in each SHAPE pattern (resolution cutoff 4.0 Å).
[8]: Wilcoxon signed-rank test for C2′-endo populations between positions involved in each SHAPE pattern (resolution cutoff 3.0 Å).

Supplementary Table 5 | Performances of SHAPELoop and other prediction tools on the CE SHAPE dataset

RNA	SHAPELoop		MFE+SHAPE		MEA+SHAPE		MFE		RME		SeqFold	
	Sensitivity	PPV	Sensitivity	PPV	Sensitivity	PPV	Sensitivity	PPV	Sensitivity	PPV	Sensitivity	PPV
16S rRNA domain 1	0.80	0.77	0.80	0.78	0.81	0.77	0.55	0.55	0.79	0.78	0.65	0.70
16S rRNA domain 2	0.82	0.78	0.80	0.73	0.80	0.73	0.79	0.72	0.79	0.75	0.70	0.66
16S rRNA domain 3	0.89	0.83	0.91	0.83	0.91	0.84	0.30	0.28	0.90	0.84	0.82	0.88
16S rRNA domain 4	0.90	0.90	0.89	0.89	0.89	0.91	0.74	0.72	0.85	0.93	0.73	0.83
23S rRNA domain 1	0.54	0.51	0.53	0.51	0.55	0.54	0.55	0.51	0.54	0.53	0.47	0.50
23S rRNA domain 2	0.78	0.76	0.78	0.75	0.77	0.75	0.80	0.77	0.81	0.82	0.77	0.78
23S rRNA domain 3	0.81	0.81	0.81	0.81	0.81	0.81	0.47	0.46	0.84	0.80	0.65	0.68
23S rRNA domain 4	0.69	0.66	0.54	0.51	0.70	0.69	0.69	0.56	0.58	0.53	0.61	0.57
23S rRNA domain 5	0.64	0.62	0.65	0.62	0.66	0.63	0.48	0.46	0.63	0.62	0.50	0.53
23S rRNA domain 6	0.92	0.84	0.87	0.82	0.91	0.84	0.68	0.57	0.93	0.84	0.74	0.63
5S rRNA	0.94	0.78	0.94	0.78	0.26	0.23	0.26	0.22	0.94	0.80	0.46	0.44
adenine riboswitch	1.00	1.00	1.00	1.00	1.00	1.00	1.00	1.00	1.00	1.00	1.00	1.00
cyclic di-GMP riboswitch	1.00	0.88	0.80	0.74	0.80	0.80	0.80	0.74	0.80	0.80	0.81	0.67
glycine riboswitch	0.97	0.92	0.93	0.84	0.93	0.84	0.70	0.61	0.80	0.73	0.65	0.90
domain II of the HCV IRES[1]	0.83	1.00	0.77	1.00	0.77	1.00	0.77	1.00	0.77	1.00	0.57	1.00
P546 domain, group I intron	0.91	0.96	0.91	0.96	0.93	0.98	0.41	0.44	0.91	0.96	0.59	0.94
tRNA^{Asp}	0.91	0.95	0.91	0.95	0.91	0.95	0.91	0.95	0.73	0.94	0.64	0.65
tRNA^{phe}	1.00	0.96	0.75	0.71	0.75	0.71	0.95	0.95	1.00	0.95	0.41	0.51
P4-P6 domain, group I intron	0.96	0.91	0.96	0.89	0.96	0.90	0.98	0.91	0.96	0.86	0.90	0.84
**Mean**	0.86	0.83	0.82	0.80	0.80	0.79	0.68	0.65	0.82	0.81	0.67	0.72
**Median**	0.90	0.84	0.81	0.81	0.81	0.81	0.70	0.61	0.81	0.82	0.65	0.68
**Standard error**	0.12	0.13	0.13	0.14	0.17	0.18	0.21	0.23	0.13	0.14	0.15	0.17
**Confidence interval[2]**	[0.80, 0.91]	[0.77, 0.89]	[0.76, 0.87]	[0.73, 0.86]	[0.71, 0.86]	[0.70, 0.86]	[0.58, 0.77]	[0.55, 0.76]	[0.76, 0.88]	[0.75, 0.87]	[0.60, 0.74]	[0.65, 0.80]
**Number of compared RNAs**	19	19	19	19	19	19	19	19	19	19	19	19

[1]: Internal ribosome entry sequence.
[2]: 95% confidence intervals calculated by bootstrap percentile method following a previous work (11).

Supplementary Table 6 | Performances of SHAPELoop and other prediction tools on the SHAPE-MaP dataset

RNA	MFE+CE SHAPE		MFE+SHAPE-MaP		SHAPELoop (SHAPE-MaP)	
	Sensitivity	PPV	Sensitivity	PPV	Sensitivity	PPV
16S rRNA domain 1	0.80	0.78	**0.87**	**0.86**	**0.87**	0.85
16S rRNA domain 2	**0.80**	0.73	0.79	**0.74**	**0.80**	**0.74**
16S rRNA domain 3	**0.91**	**0.83**	0.78	0.77	0.77	0.77
16S rRNA domain 4	**0.89**	**0.89**	0.74	0.69	0.74	0.70
23S rRNA domain 1	0.53	0.51	0.53	**0.52**	**0.54**	**0.52**
23S rRNA domain 2	0.78	0.75	**0.81**	**0.80**	0.80	0.79
23S rRNA domain 3	**0.81**	**0.81**	0.66	0.63	0.76	0.76
23S rRNA domain 4	0.54	0.51	0.75	0.68	**0.77**	**0.71**
23S rRNA domain 5	**0.65**	**0.62**	0.63	0.59	0.60	0.58
23S rRNA domain 6	**0.87**	**0.82**	0.67	0.59	0.79	0.75
5s rRNA	0.94	0.78	0.94	0.82	**1.00**	**1.00**
P4-P6 domain, group I intron	0.96	0.89	**0.98**	**0.92**	**0.98**	**0.92**
domain II of the HCV IRES[1]	**0.77**	**1.00**	**0.77**	**1.00**	**0.77**	0.96
**16S rRNA (mean)**	**0.85**	**0.81**	0.79	0.76	0.79	0.77
**23S rRNA (mean)**	0.70	0.67	0.67	0.63	**0.71**	**0.69**
**Mean**	**0.79**	0.76	0.76	0.74	0.78	**0.77**
**Median**	**0.80**	**0.78**	0.77	0.74	0.77	0.76
**Winner counts**	**7**	**6**	4	**6**	**7**	5

[1]: Internal ribosome entry sequence.
The best sensitivity and PPV among all tested algorithms are shown in bold.

Supplementary references

1. Dube, A., Bolduc, F., Bisaillon, M. and Perreault, J.P. (2011) Mapping studies of the Peach latent mosaic viroid reveal novel structural features. *Mol. Plant. Pathol.*, **12**, 688–701.
2. Jucker, F.M., Heus, H.A., Yip, P.F., Moors, E.H. and Pardi, A. (1996) A network of heterogeneous hydrogen bonds in GNRA tetraloops. *J. Mol. Biol.*, **264**, 968–980.
3. Allain, F.H. and Varani, G. (1995) Structure of the P1 helix from group I self-splicing introns. *J. Mol. Biol.*, **250**, 333–353.
4. Deigan, K.E., Li, T.W., Mathews, D.H. and Weeks, K.M. (2009) Accurate SHAPE-directed RNA structure determination. *Proc. Natl. Acad. Sci. U. S. A.*, **106**, 97–102.
5. Cordero, P., Lucks, J.B. and Das, R. (2012) An RNA Mapping DataBase for curating RNA structure mapping experiments. *Bioinformatics*, **28**, 3006–3008.
6. Michel, F. and Westhof, E. (1990) Modelling of the three-dimensional architecture of group I catalytic introns based on comparative sequence analysis. *J. Mol. Biol.*, **216**, 585–610.
7. Duncan, C.D. and Weeks, K.M. (2008) SHAPE analysis of long-range interactions reveals extensive and thermodynamically preferred misfolding in a fragile group I intron RNA. *Biochemistry*, **47**, 8504–8513.
8. Westhof, E., Dumas, P. and Moras, D. (1985) Crystallographic refinement of yeast aspartic acid transfer RNA. *J. Mol. Biol.*, **184**, 119–145.
9. Wilkinson, K.A., Merino, E.J. and Weeks, K.M. (2005) RNA SHAPE chemistry reveals nonhierarchical interactions dominate equilibrium structural transitions in tRNA(Asp) transcripts. *J. Am. Chem. Soc.*, **127**, 4659–4667.
10. Lukavsky, P.J., Kim, I., Otto, G.A. and Puglisi, J.D. (2003) Structure of HCV IRES domain II determined by NMR. *Nat. Struct. Biol.*, **10**, 1033–1038.
11. Hajiaghayi, M., Condon, A. and Hoos, H.H. (2012) Analysis of energy-based algorithms for RNA secondary structure prediction. *BMC Bioinf.*, **13**, 22.
